# Vδ2 T-cell engagers bivalent for Vδ2-TCR binding provide anti-tumor immunity and support robust Vγ9Vδ2 T-cell expansion

**DOI:** 10.3389/fonc.2024.1474007

**Published:** 2024-10-18

**Authors:** Lisa A. King, Milon de Jong, Myrthe Veth, David Lutje Hulsik, Parsa Yousefi, Victoria Iglesias-Guimarais, Pauline M. van Helden, Tanja D. de Gruijl, Hans J. van der Vliet

**Affiliations:** ^1^ Department of Medical Oncology, Amsterdam University Medical Center (UMC), Vrije Universiteit Amsterdam, Amsterdam, Netherlands; ^2^ Cancer Center Amsterdam, Amsterdam, Netherlands; ^3^ Amsterdam Institute for Infection and Immunity, Amsterdam, Netherlands; ^4^ Lava Therapeutics NV, Utrecht, Netherlands

**Keywords:** Vγ9Vδ2 T-cells, bispecific T-cell engager, single domain antibody, expansion, immunotherapy, cancer

## Abstract

**Background:**

Vγ9Vδ2 T-cells are antitumor immune effector cells that can detect metabolic dysregulation in cancer cells through phosphoantigen-induced conformational changes in the butyrophilin (BTN) 2A1/3A1 complex. In order to clinically exploit the anticancer properties of Vγ9Vδ2 T-cells, various approaches have been studied including phosphoantigen stimulation, agonistic BTN3A-specific antibodies, adoptive transfer of expanded Vγ9Vδ2 T-cells, and more recently bispecific antibodies. While Vγ9Vδ2 T-cells constitute a sizeable population, typically making up ~1-10% of the total T cell population, lower numbers have been observed with increasing age and in the context of disease.

**Methods:**

We evaluated whether bivalent single domain antibodies (VHHs) that link Vδ2-TCR specific VHHs with different affinities could support Vγ9Vδ2 T-cell expansion and could be incorporated in a bispecific engager format when additionally linked to a tumor antigen specific VHH.

**Results:**

Bivalent VHHs that link a high and low affinity Vδ2-TCR specific VHH can support Vγ9Vδ2 T-cell expansion. The majority of Vγ9Vδ2 T-cells that expanded following exposure to these bivalent VHHs had an effector or central memory phenotype and expressed relatively low levels of PD-1. Bispecific engagers that incorporated the bivalent Vδ2-TCR specific VHH as well as a tumor antigen specific VHH triggered antitumor effector functions and supported expansion of Vγ9Vδ2 T-cells *in vitro* and in an *in vivo* model in NOG-hIL-15 mice.

**Conclusion:**

By enhancing the number of Vγ9Vδ2 T-cells available to exert antitumor effector functions, these novel Vδ2-bivalent bispecific T cell engagers may promote the overall efficacy of bispecific Vγ9Vδ2 T-cell engagement, particularly in patients with relatively low levels of Vγ9Vδ2 T-cells.

## Introduction

Vγ9Vδ2 T-cells constitute a homogeneous unconventional T-cell population that orchestrates both innate and adaptive immunity (1). Vγ9Vδ2 T-cells recognize target cells in an HLA-independent manner through phosphoantigen (pAg)-induced conformational changes in the butyrophilin (BTN) 2A1/3A1 complex (2, 3). pAg are metabolites that accumulate intracellularly due to dysregulation of the mevalonate pathway during cellular stress, caused by such processes as infection or malignant transformation, or upon exposure to aminobisphosphonates (N-BP) (4). Following activation, Vγ9Vδ2 T-cells can cross-present antigens (5–7), produce proinflammatory cytokines and chemokines, and induce cytotoxicity in a wide range of malignancies via membrane bound TRAIL and Fas ligand or through granzyme B/perforin release (8).

Although Vγ9Vδ2 T-cells make up ~1-10% of CD3^+^ T-cells in the circulation, their numbers can be reduced with increasing age and in the context of disease (9–14). Notably, the presence of γδ T-cells within tumor-infiltrating immune cells (15) and the relative abundance of specifically tumor-infiltrating Vγ9Vδ2 T-cells correlate to improved patient outcome (16, 17). Several therapeutic approaches designed to exploit the antitumor properties of Vγ9Vδ2 T-cells were explored. N-BPs such as pamidronate and zoledronate, as well as synthetic pAg analogues like bromohydrin pyrophosphate (BrHPP), were tested, either alone or in combination with IL-2, to activate Vγ9Vδ2 T-cells *in vivo* or *ex vivo* followed by adoptive cell therapy (ACT) in various malignancies. While these strategies were overall well-tolerated and safe, and resulted in increased frequencies of circulating Vγ9Vδ2 T-cells across different types of cancer patients, only a minority of patients participating in these clinical trials exhibited significant signs of anti-tumor efficacy (8, 18, 19).

A novel approach based on Vγ9Vδ2 T-cell stimulation using an agonistic BTN3A-specific monoclonal antibody alone or in combination with IL-2 is currently being evaluated in a phase 2 clinical trial in advanced-stage cancer patients (20, 21). In addition, multiple efforts are ongoing to incorporate tumor-targeting moieties in Vγ9Vδ2 T-cell based cancer therapies, including the use of chimeric antigen receptors (CAR) and bispecific antibodies. Multiple studies, both preclinical and clinical, have reported promising antitumor activity upon administration of CAR-modified or monoclonal antibody conjugated expanded (Vδ1 or Vδ2) γδ T cells (22–27). Adoptive cell therapy, and in particular allogeneic CAR T-cell therapy, poses several challenges, not only related to the laborious nature of the manufacturing process with associated high costs, but also to limitations in transduction efficacy, product success rate, and the requirement of lymphodepleting preconditioning chemotherapy regimens to enhance their persistence (28, 29). Bispecific T-cell engagers (bsTCEs), which consist of linked antibody binding domains directed against a tumor associated antigen and a T-cell specific receptor, potentially provide a relatively cost-effective *off-the-shelf* approach to redirect endogenous T-cells to tumors (30). We and others have developed bispecific engagers to direct Vγ9Vδ2 T-cells to tumors and demonstrated that these have the potential to induce robust lysis of various malignancies in a tumor associated antigen restricted fashion (31–37). Importantly, early signs of potential anti-tumor efficacy were also noted during phase 1 dose escalation in patients treated with bispecific Vγ9Vδ2 T-cell engagers with a high-affinity Vδ2-TCR specific VHH (38, 39). One can envision that the antitumor efficacy of these bispecific Vγ9Vδ2 T-cell engagers can be further promoted by enhancing the number of Vγ9Vδ2 T-cells available to exert antitumor effector functions *in vivo*. To this end, we explored whether a Vδ2-TCR binding arm that consisted of two Vδ2-TCR specific VHHs could combine antitumor activity with expansion of Vγ9Vδ2 T-cells.

Here, we report that bispecific Vγ9Vδ2 T-cell engagers that combine a tumor antigen-specific single domain antibody (VHH) with a bivalent VHH, that links a high and low affinity Vδ2-TCR specific VHH, trigger antitumor effector functions and support expansion of Vγ9Vδ2 T-cells *in vitro* and *in vivo*. By enhancing the number of Vγ9Vδ2 T-cells available to exert antitumor effector functions in the tumor microenvironment, these novel Vδ2-bivalent bsTCEs may promote the overall efficacy of bispecific Vγ9Vδ2 T-cell engagement, particularly in patients with relatively low levels of Vγ9Vδ2 T-cells.

## Materials and methods

### Tumor cell lines

SW480 tumor cells (EGFR^+^) were obtained from American Type Culture Collection (ATCC, CCL-228) and maintained in Dulbecco’s Modified Eagle Medium (DMEM, 1965-039, Gibco) supplemented with 10% (v/v) fetal calf serum (FCS, 04-007-1A, Biological Industries), 0.05 mM β‐mercaptoethanol (β‐ME, 200-646-6, Merck), and 100 IU/ml sodium penicillin, 100 μg/ml streptomycin sulphate, and 2.0 mM L‐glutamine (PSG, 10378-016, Life technologies). 22Rv1 tumor cells (PSMA^+^) were obtained from the European Collection of Authenticated Cell Cultures (ECACC, 5092802) and MM.1s tumor cells (kind gift from R. Groen, Amsterdam UMC, Vrije Universiteit, Amsterdam, the Netherlands) were transfected with CD1d as described previously (40) (referred to as MM.1s.CD1d). The 22Rv1 and MM.1s tumor cell lines were maintained in Roswell Park Memorial Institute 1640 (RPMI-1640, 22400089, Gibco) medium supplemented with 10% FCS, β-ME, and PSG. Tumor cell lines were tested regularly for *Mycoplasma* using PCR.

### PBMC isolation and Vγ9Vδ2 T-cell cultures

Healthy donor- and cancer patient peripheral blood mononuclear cells (PBMC) were isolated from whole blood by density gradient centrifugation using Lymphoprep™ (AXI-1114547, Fresenius). Healthy donor blood samples were obtained under written informed consent from Sanquin (Amsterdam, the Netherlands). Blood samples from cancer patients were obtained after approval by the institutional review board (medical ethical committee Amsterdam UMC, location VUmc) and written informed consent was obtained from all the participants from the Amsterdam UMC (location VUmc, Amsterdam, the Netherlands). Expanded Vγ9Vδ2 T-cells were generated from healthy donor PBMC-derived Vγ9Vδ2 T-cells as described before (33). In short, Vδ2^+^ T cells were isolated from healthy donor PBMC using magnetic bead sorting using FITC-conjugated Vδ2 antibody (**Supplementary Table S1**) in combination with anti-mouse IgG microbeads (130-048-401, Miltenyi Biotec). Purified Vγ9Vδ2 T-cells were stimulated weekly with irradiated feeder mix consisting of healthy donor PBMC (1 x 10^6^ cells/ml), JY cells (1 x 10^5^ cells/mL, 94022533, ECACC), IL-7 (10 U/mL, 207-IL-025, R&D Systems), IL-15 (10 ng/mL, 34-8159-85, eBioscience), and PHA (50 ng/mL, R30852801, Thermo Fisher Scientific). Purity of Vγ9Vδ2 T-cells used in experiments was ≥95%. All functional experiments were performed in RPMI medium supplemented with 10% FCS, β-ME, and PSG. Cell cultures were kept at 37˚C in a humidified atmosphere containing 5% CO_2_.

### Design, production and purification of Vδ2-TCR-specific constructs

Human Vδ2-TCR specific llama-derived VHHs were generated and screened as previously described (41). In short, two *Lama glamas* were immunized with human Vγ9Vδ2 T-cells and Vδ2-TCR-specific VHHs were selected using phage display followed by confirmation of specific binding to Vδ2^+^ T cells by flow cytometry. Bivalent constructs were generated by linking two monovalent Vδ2-TCR-specific VHHs with low (VHH-5C7, Kd ~350nM), intermediate (VHH-5D3, Kd ~21nM) or high (VHH-6H4, Kd ~0.4nM) affinity in various combinations using a Gly4Ser amino acid based linker [(G4S)_2_; referred to as 10 amino acid (AA) linker], resulting in the following bivalent constructs: 6H4-(G4S)_2_-5C7, 5C7-(G4S)_2_-6H4, 6H4-(G4S)_2_-6H4, 5D3-(G4S)_2_-6H4, 6H4-(G4S)_2_-5D3, 5D3-(G4S)_2_-5D3, 5D3-(G4S)_2_-5C7, 5C7-(G4S)_2_-5D3, 5C7-(G4S)_2_-5C7. The bivalent construct 6H4-5C7 was generated with multiple linker lengths; G4S, (G4S)_2_ or (G4S)_4_ (referred to as 5, 10 or 20 AA linker). Purified protein was produced by ImmunoPrecise Antibodies (IPA, Utrecht, The Netherlands) using DNA transfected HEK293E cells and rmp Protein A affinity chromatography followed by preparative size exclusion. Proteins used were >95% pure and monomeric.

Three different tumor associated antigen (TAA) specific VHHs [i.e. directed against EGFR (VHH-7D12 (42)], PSMA [VHH-JVZ-007 (43)] or CD1d [VHH-1D12 (44)] were linked with a G4S linker to the N-terminus of 6H4-5C7 (termed Vδ2^hi-lo^): EGFR-G4S-6H4-(G4S)_2_-5C7, PSMA-G4S-6H4-(G4S)_2_-5C7 and CD1d-G4S-6H4-(G4S)_2_-5C7 (referred to as EGFR-Vδ2^hi-lo^ bsVHH, PSMA-Vδ2^hi-lo^ bsVHH and CD1d-Vδ2^hi-lo^ bsVHH). Next, Fc domain and anti-albumin VHH containing molecules were created to allow for *in vivo* plasma half-life extension. For the Fc domain, knobs-into-holes technology was used for heterodimerization (HC1, knob mutation T366W and HC2, hole mutations T366S, L368A, Y407V), LFLE mutations were included to silence the Fc domain except for the neonatal Fc receptor (FcRn) (L234F and L235E silencing, EU numbering), a Cys220 deletion was incorporated to avoid an unpaired cysteine and a modified hinge was used (HC1: 5C7-G4S-6H4-hinge-Fc paired with HC2: EGFR-hinge-Fc, PSMA-hinge-Fc or CD1d-hinge-Fc; referred to as EGFR-Vδ2^hi-lo^ bsVHH-Fc, PSMA-Vδ2^hi-lo^ bsVHH-Fc and CD1d-Vδ2^hi-lo^ bsVHH-Fc. Expression of these tumor targeting VHHs was performed via pcDNA3.1(+) (100µg, Genscript) transfection in Expi293F cells (A14527, Gibco) using the ExpiFectamine 293 Transfection Kit (A14524, Gibco). Proteins were purified using rmp Protein A Sepharose Fast Flow resin (17-5138-03, GE healthcare) (EGFR-Vδ2^hi-lo^ bsVHH, PSMA-Vδ2^hi-lo^ bsVHH and CD1d-Vδ2^hi-lo^ bsVHH) or CaptureSelect C-tagXL Affinity Matrix (2943072050, Thermo Scientific) (EGFR-Vδ2^hi-lo^ bsVHH-Fc, PSMA-Vδ2^hi-lo^ bsVHH-Fc or CD1d-Vδ2^hi-lo^ bsVHH-Fc). Quality control was done using size exclusion ultra performance liquid chromatography. For fusion to an anti-albumin (Alb) VHH (45), the following construct was generated: EGFR-(G4S)_2_-Alb-(G4S)_2_-6H4-G4S-5C7 (referred to as EGFR-Vδ2^hi-lo^ bsVHH-albumin; illustrated in **Supplementary Figure S4A**). Purified protein for the construct was produced by IPA as described above. Proteins used were >95% pure and >94% heterodimer shown by native mass spectrometry analysis. Lastly, bispecific engagers containing a monovalent Vδ2-TCR-specific VHH linked with a TAA-specific VHH, i.e. EGFR-G4S-5C8, PSMA-G4S-5C8 and CD1d-G4S-5C8 (referred to as EGFR-Vδ2 bsVHH, PSMA-Vδ2 bsVHH and CD1d-Vδ2 bsVHH), were generated as described before (31, 32) and are illustrated in **Supplementary Figure S3A**.

### Flow cytometry

Cells were resuspended in PBS (1073508600, Fresenius Kabi) supplemented with 0.5% bovine serum albumin (M090001/03, Fisher Scientific) and 20 μg/ml NaN3 (247-852-1, Merck) and incubated with fluorochrome-labeled monoclonal antibodies and viability dyes (**Supplementary Table S1**) for 30 min at 4°C. Unbound fluorochrome-labeled antibodies were washed away. Cells were analyzed using the FACS LSRFortessa XL-20 (BD Biosciences) and data analysis was performed using FlowJo v10.8.1 (BD Biosciences).

### Target cell binding by Vδ2-TCR specific constructs

Binding of the various bivalent and bispecific VHH constructs to Vδ2, EGFR, PSMA or CD1d was evaluated using flow cytometry or ELISA, as indicated. For flow cytometry analysis, Vγ9Vδ2 T-cells, SW480, 22Rv1 or MM.1s.CD1d tumor cells were incubated with a concentration range of the constructs for 45 min at 4°C, followed by extensive washing (5x) and incubation for 30 min at 4°C with FITC-labeled rabbit-anti-llama polyclonal Ab (**Supplementary Table S1**). For the ELISA, wells of a clear bottom F96-well Maxisorp plate (439454, Nunc) were coated overnight with human Gamma9Delta2 TCR (0.5 µg/mL, produced by IPA). Wells were blocked with 2% BSA (A2153, Sigma) for 60 min, and test samples were incubated for 120 min at room temperature. The rabbit anti-camelid VHH cocktail (60-min incubation, 19L002038, GenScript) and swine anti-rabbit immunoglobulins HRP (60-min incubation, 41289300, Dako) were used for detection and 3,3’,5,5’-Tetramethylbenzidine (TMB, Life Technologies, #SB02) was used as a substrate for color development. The absorbance (450 nm) was measured using a plate reader (Molecular Devices, iD5) and the data analysis was done using SoftMax Pro software v 7.1.

### Assessment of Vγ9Vδ2 T-cell frequency, expansion and phenotype using healthy donor and cancer patient PBMC

Vγ9Vδ2 T-cell percentages in healthy donor and cancer patient-derived PBMC (patient characteristics provided in **Supplementary Table S2**) were assessed using flow cytometry using fluorescently-labelled antibodies against CD3, Vγ9, Vδ2 (**Supplementary Table S1**). The capacity of the various bivalent and bispecific VHH constructs to induce Vγ9Vδ2 T-cell expansion was assessed by incubating healthy donor or cancer patient-derived PBMC (patient characteristics provided in **Supplementary Table S3**) with indicated concentrations of the bivalent or bispecific constructs, 10 µM pamidronate disodium salt hydrate (referred to as pamidronate, P2371-10MG, Sigma) (positive control) or medium (negative control). Twenty-four hrs later, 100 IU/ml IL-2 (Proleukin, Clinigen) was added to all conditions (including medium, termed IL-2 control). Fold expansion and enrichment of Vγ9Vδ2 T-cells (compared to baseline) were assessed using flow cytometry at the end of the 8-day culture period using fluorescently-labelled antibodies against CD3, Vγ9, Vδ2 and 123counting eBeads™ (01-1234-42, Thermofisher). Depending on the experiment, 7-AAD (A9400-1MG, Sigma), fixable viability dyes eFluor™ 506 or eFluor™ 780 (**Supplementary Table S1**) were used to identify viable cells. The phenotype of expanded Vγ9Vδ2 T-cells was determined using fluorescently-labelled antibodies against CD27, CD45RA, CD25, CD69, HLA-DR, DNAM-1, NKG2D, NKG2A, PD-1, CTLA-4 and TIGIT (see **Supplementary Table S1** for clones and fluorophores).

### Assessment of Vγ9Vδ2 T-cell degranulation and target cell lysis

Vγ9Vδ2 T-cell degranulation and subsequent tumor cell lysis were assessed by flow cytometry. Expanded healthy donor-derived Vγ9Vδ2 T-cells were incubated with SW480, 22Rv1 or MM.1s.CD1d tumor cells (1:1 E:T ratio) for 24hrs with a concentration range of the TAA-Vδ2^hi-lo^ bsVHH with or without Fc domain or anti-albumin-VHH, or medium control. Degranulation and activation of Vγ9Vδ2 T-cells was assessed using fluorescently-labelled antibodies against CD3, Vγ9, Vδ2, CD25 and CD107a (**Supplementary Table S1**) and tumor cell lysis was determined using 7-AAD and 123counting eBeads^TM.^ To assess degranulation and tumor cell lysis of 8-day expanded Vγ9Vδ2 T-cells in healthy donor derived PBMC, γδ T-cells were isolated from the PBMC cultures using the untouched human TCR γ/δ^+^ T-cell isolation kit (130-092-892, Miltenyi Biotec) and used as described above in the presence or absence of the respective TAA-Vδ2^hi-lo^ bsVHH or TAA-Vδ2^hi-lo^ bsVHH-Fc engagers. Supernatants were collected and stored at -20°C until further analysis for IL-2, TNF and IFN-γ secretion using the human Th1/Th2/Th17 cytometric bead array (CBA) kit (560484, BD). CBA data were analyzed with FCAP Array software v3.0 (BD).

### *In vivo* mouse study

Sub-lethally irradiated (1.75 Gy on day 0) NOD.Cg-Prkdcscid Il2rgtm1Sug/JicTac (NOG)-hIL-15 mice (8.5 weeks old, #13683-F Taconic Biosciences) were kept under pathogen-free conditions (Laboratory Animal Center of the Netherlands Cancer Institute, Amsterdam, The Netherlands) and used for evaluation of *in vivo* expansion of Vγ9Vδ2 T-cells in PBMC. This *in vivo* experiment was approved by the Animal Welfare Committee of the Netherlands Cancer Institute and was performed in accordance with national guidelines. Mice were randomized into 2 groups (n=4/group) and intravenously (i.v.) inoculated with 10x10^6^ healthy human donor-derived PBMC (n=1 healthy donor with 0.89% Vγ9Vδ2 T-cells of total CD3^+^ T-cells) on day 0. PBS or the CD1d-Vδ2^hi-lo^ bsVHH (0.5 mg/kg) were intraperitoneally (i.p.) administered on days 0 and 4. Blood (day 1 and 8) and lungs, spleens and livers (day 8) were collected and prepared for flow cytometry analysis. Erythrocytes were lysed using lysis buffer (0.16 M NH_4_Cl; 76050139.1000, Boom and 0.011 M KHCO_3_; 104854, Merck). Lungs were dissociated while stirring in a flask for 45 min at 37°C using Iscove’s Modified Dulbecco’s Medium (IMDM) supplemented with 5% FCS, PSG, 0.1% DNAse I (10104159001, Roche) and 0.14% collagenase A (10103586001, Roche). Spleens and livers were dissociated using a 100 µM cell strainer (352360, Falcon) and a syringe plunger, and dissociated cells were washed with PBS. Blood and tissue-derived single cell suspensions were used for flow cytometry analysis using a combination of 7-AAD, fluorescently-labelled antibodies against CD45 (mouse), CD45 (human), CD3, Vγ9, Vδ2, CD27, CD45RA and 123counting eBeads™ for Vγ9Vδ2 T-cell analysis (**Supplementary Table S1**).

### Statistical analysis

Analysis were performed using Prism v.9.1.0 (GraphPad Software). Data were analyzed using unpaired or paired *t* test, one-way analysis of variance (ANOVA) with Tukey’s comparisons test, or two-way ANOVA with Tukey’s multiple comparisons test, as appropriate. Binding curves and dose–response curves with EC_50_ values were calculated using nonlinear regression analysis. To calculate the correlation between Vγ9Vδ2 T-cell frequency and donor age, Spearman’s rank correlation analysis was used. *P=*< 0.05 was considered significant and showed with asterisks: *P=<* 0.05: *, *P=*< 0.01: **, *P=*< 0.001: ***, *P=*< 0.0001: ****.

## Results

### Vγ9Vδ2 T-cell frequencies in peripheral blood of healthy adult donors and adult cancer patients

Given that the Vγ9Vδ2 T-cell frequency in peripheral blood has been reported to decline with age and can be reduced due to underlying malignancies (9–14), we assessed the frequency of Vγ9Vδ2 T-cells in peripheral blood of healthy donors [n=121; 68 females, 53 males, mean age (range): 41 (18-77)] and cancer patients with various solid tumors (n=91; see **Supplementary Table S2** for patient characteristics). As shown in **Supplementary Figure S1A**, the Vγ9Vδ2 T-cell frequency varied substantially between individuals and was significantly higher in healthy donor compared to cancer patient PBMC (3.5 ± 0.3% versus 2.2 ± 0.4% of total T cells; mean ± SEM, *P* = 0.01). In healthy female and male donors, the Vγ9Vδ2 T-cell frequency negatively correlated with donor age (**Supplementary Figure S1B**). This negative correlation between Vγ9Vδ2 T-cell frequency and age was not (statistically significantly) observed in cancer patients, though the population of adult patients with solid tumors evaluated in our analysis was generally skewed towards older age (66 ± 0.9 yr versus 40 ± 1.5 yr in healthy donors; mean ± SEM; **Supplementary Figure S1C**). Of interest, the frequency of Vγ9Vδ2 T-cells was comparable in cancer patients and healthy donors with age ≥ 39 yr (Vγ9Vδ2 T-cell frequency 2.0 ± 1.9% versus 2.3 ± 3.3% of total T cells in healthy donors ≥ 39 yr and cancer patients ≥ 39 yr respectively; mean ± SEM; **Supplementary Figure S1D**), suggesting that the Vγ9Vδ2 T-cell frequencies in peripheral blood of cancer patients are predominantly influenced by age, rather than by the underlying malignancy.

### A bivalent Vδ2-TCR specific VHH that combines high and low affinity Vδ2 binding domains supports Vγ9Vδ2 T-cell expansion

**Figure 1:**
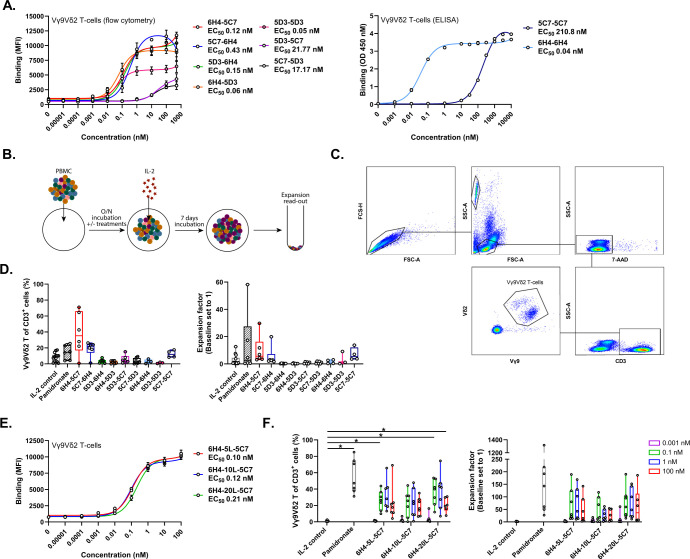
Bivalent Vδ2-TCR-specific VHHs support enrichment and expansion of Vγ9Vδ2 T-cells. **(A)** Binding of bivalent Vδ2-VHHs to Vγ9Vδ2 T-cells assessed using flow cytometry (n=3) or ELISA (n=2). **(B)** Overview of experimental design to assess expansion. **(C)** Representative gating strategy to asses enriched and expanded Vγ9Vδ2 T-cells within PBMC. **(D)** Enrichment (left panel) and fold expansion (right panel) of Vγ9Vδ2 T-cells during an 8 day culture of healthy donor PBMC in the presence or absence of 1 nM bivalent Vδ2-VHHs or 10 µM pamidronate (n=3-6). **(E)** Binding of the 6H4-5C7 bivalent VHH with either a 5, 10 or 20 AA linker to Vγ9Vδ2 T-cells (n=3). **(F)** Enrichment (left panel) and fold expansion (right panel) of Vγ9Vδ2 T-cells during an 8 day culture of healthy donor PBMC in the presence or absence of 0.001, 0.1, 1 or 100 nM of the 6H4-5C7 bivalent VHH with either 5, 10 or 20 AA linker or 10 µM pamidronate. Data in **(A)** (left panel) and **(D–F)** assessed using flow cytometry. Data in A (right panel) assessed using ELISA. Data represent mean and SEM **(A, E)** or individual data-points are indicated using open circles and box and whisker plots indicate the median, 25th to 75th percentiles and minimum to maximum **(D, F)**. Two-way ANOVA with Tukey’s multiple comparisons test **(F)** were used for statistical analysis; *P*=< 0.05: *.

As TCR cross-linking, which is not achieved with monovalent TCR binding, initiates signaling typically resulting in activation, differentiation, cytokine production and proliferation, we evaluated whether linkage of two Vδ2-TCR specific VHHs could promote Vγ9Vδ2 T-cell expansion. Three Vδ2-TCR specific VHHs were selected based on affinity (i.e. VHH-5C7, Kd ~350 nM; VHH-5D3, Kd ~21 nM; and VHH-6H4, Kd ~0.4 nM) (ref (41) and not shown), and differentially combined and linked using a 10 AA linker into nine different bivalent VHHs. Binding of the various bivalent Vδ2-VHHs to Vγ9Vδ2 T-cells was assessed using flow cytometry or ELISA and showed that the binding EC_50_ was comparable for compounds that contained a high affinity VHH linked to either a low or intermediate affinity VHH or two intermediate-binding VHHs (EC_50_ = 0.05-0.43 nM) (**Figure 1A**). For the high and low affinity VHH combination, N-terminal positioning of the high affinity VHH-6H4 resulted in a lower EC_50_ than when this VHH was positioned C-terminally. Compounds consisting of an intermediate and a low affinity VHH bound with higher EC_50_ values of 17-22 nM. The binding EC_50_ of the linked low affinity VHHs could not be reliably assessed using flow cytometry and was therefore determined using ELISA, resulting in a value of 211 nM (**Figure 1A** right panel). To assess whether the bivalent Vδ2-VHHs could support Vγ9Vδ2 T-cell expansion, healthy donor derived PBMC were cultured for 8 days in the presence or absence of 1 nM (optimal concentration across the compounds as determined by titration, data not shown) of the bivalent Vδ2-VHHs or 10 µM pamidronate. After 24 hrs, 100 IU/ml of recombinant human (rh) IL-2 was added to the cultures (including the medium control, termed IL-2 control). **Figure 1B** illustrates the design of the experiment and **Figure 1C** shows a representative gating strategy for the assessment of the Vγ9Vδ2 T-cell frequency. As shown in **Figure 1D**, maintained or increased Vγ9Vδ2 T-cell numbers were only observed in the conditions exposed to bivalent VHHs containing either 2 linked low affinity VHHs or the low affinity VHH linked to the high affinity VHH. When enrichment of Vγ9Vδ2 T-cells was assessed, a similar pattern was observed. Enrichment and expansion were most consistent and pronounced when PBMC were cultured with the bivalent high-low affinity 6H4-5C7 (i.e. N-terminally positioned 6H4) VHH (enrichment 35.3 ± 49.4%; median ± IQR; *P* = 0.03, and expansion 5.6 ± 12.9 fold; median ± IQR; *P* = 0.07, paired *t* tests). Most of the other bivalent Vδ2-VHHs did not support enrichment and expansion and in some cases even resulted in a reduction of Vγ9Vδ2 T-cell numbers, possibly due to fratricide. As expected, enrichment and expansion were also observed when PBMC were cultured in the presence of pamidronate. In the absence of IL-2, neither pamidronate nor bivalent Vδ2-VHHs triggered Vγ9Vδ2 T-cell expansion (**Supplementary Figure S2**). To assess the impact of different linker lengths, bivalent VHHs with the high affinity Vδ2-TCR VHH-6H4 and the low affinity Vδ2-TCR VHH-5C7 were generated with either a G4S, (G4S)_2_ or (G4S)_4_ linker (referred to as 5, 10 or 20 AA linker). As shown in **Figures 1E, F**, these bivalent (6H4-5C7) Vδ2-VHHs showed a similar binding profile (i.e. similar EC_50_ and maximum binding) and similar enrichment and expansion of Vγ9Vδ2 T-cells in the 8 day PBMC cultures. Enrichment and expansion of Vγ9Vδ2 T-cells was already observed using 0.1 nM of the bivalent (6H4-5C7) Vδ2-VHHs and not further enhanced with higher concentrations. The bivalent Vδ2-VHH with the high affinity VHH-6H4 (N-terminal) a 10 AA linker and the low affinity VHH-5C7 was selected for further experiments and termed bivalent Vδ2^hi-lo^ VHH.

### Bispecific engagers that incorporate a bivalent Vδ2-TCR-specific VHH combine Vγ9Vδ2 T-cell expansion and tumor associated antigen specific effector functions

**Figure 2:**
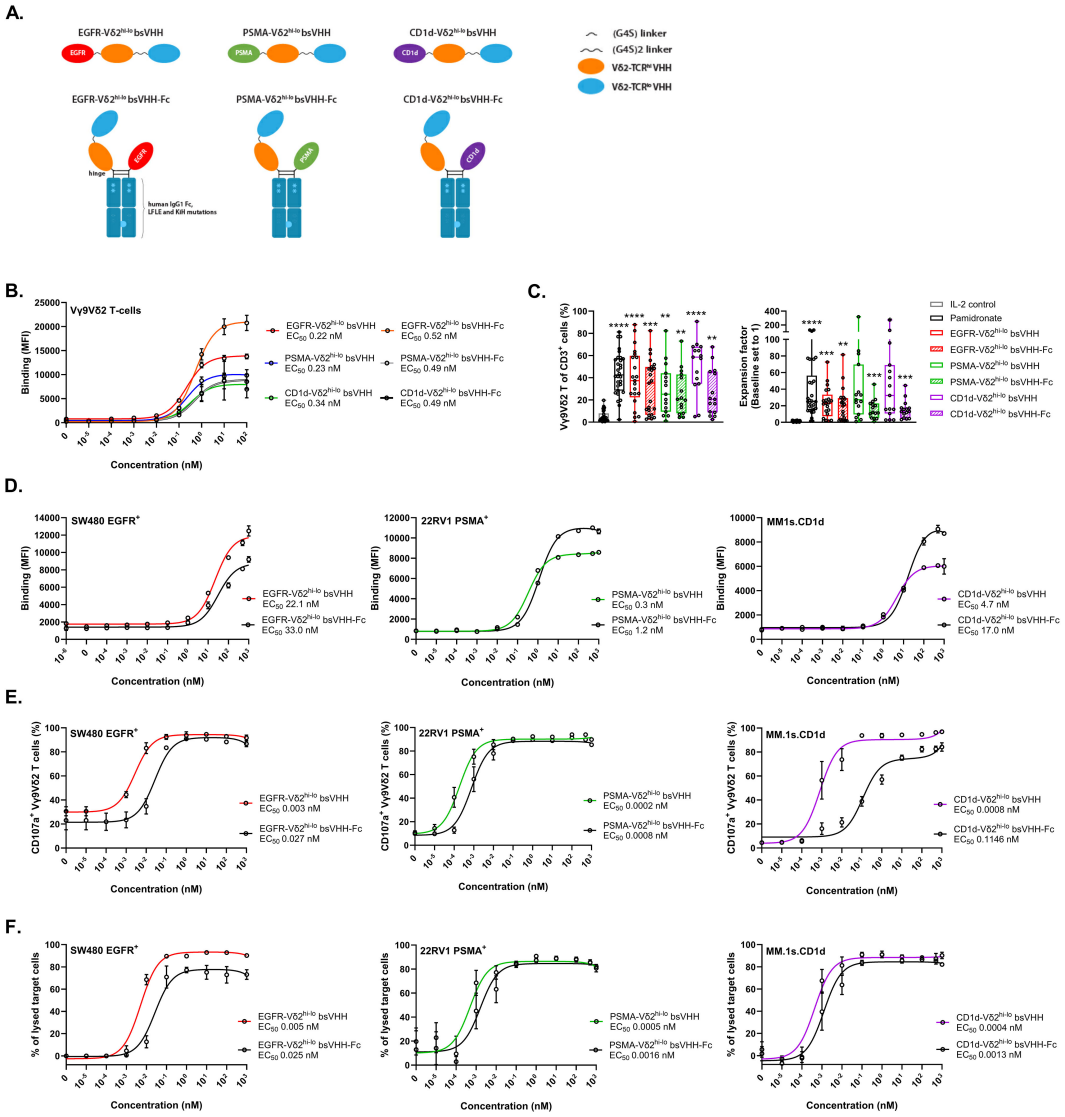
TAA-Vδ2^hi-lo^ bsVHH and TAA-Vδ2^hi-lo^ bsVHH-Fc support Vγ9Vδ2 T-cell expansion and trigger Vγ9Vδ2 T-cell degranulation and tumor cell lysis. **(A)** Illustration of the TAA-Vδ2^hi-lo^ bsVHH with and without Fc domain. **(B)** Binding of the TAA-Vδ2^hi-lo^ bsVHH with and without Fc to Vγ9Vδ2 T-cells. Data represent mean and SEM (n=3). **(C)** Enrichment (left panel) and fold expansion (right panel) of Vγ9Vδ2 T-cells during an 8 day culture of healthy donor PBMC in the presence or absence of 1 nM TAA-Vδ2^hi-lo^ bsVHH, 100nM TAA-Vδ2^hi-lo^ bsVHH-Fc or 10 µM pamidronate. Individual data-points are indicated using open circles and box and whisker plots indicate the median, 25th to 75th percentiles and minimum to maximum (n=15-30). **(D)** Binding of the TAA-Vδ2^hi-lo^ bsVHH and TAA-Vδ2^hi-lo^ bsVHH-Fc to SW480 (EGFR^+^), 22Rv1 (PSMA^+^) or MM.1s.CD1d (CD1d^+^) tumor cells. Data represent mean and SEM (n=3). **(E)** Vγ9Vδ2 T-cell CD107a expression after 24 hr co-cultures of Vγ9Vδ2 T-cells and SW480, 22Rv1 or MM.1s.CD1d tumor cells (1:1 E:T ratio) ± concentration range of the TAA-Vδ2^hi-lo^ bsVHH and TAA-Vδ2^hi-lo^ bsVHH-Fc. Data represent mean and SEM (n=3). **(F)** Lysis of SW480, 22Rv1 or MM.1s.CD1d tumor cells after 24 hr incubation with Vγ9Vδ2 T-cells (1:1 E:T ratio) ± concentration range of the TAA-Vδ2^hi-lo^ bsVHH and TAA-Vδ2^hi-lo^ bsVHH-Fc. Data represent mean and SEM (n=3-6). Data generated using flow cytometry. One-way ANOVA with Tukey’s multiple comparisons test was used for statistical analysis and asterisks are shown compared to IL-2 control; P=< 0.01: **, P=< 0.001: ***, P=< 0.0001: ****.

To explore whether a bispecific engager that includes the bivalent Vδ2^hi-lo^ VHH as well as a VHH specific for a tumor associated antigen (TAA) could combine TAA directed Vγ9Vδ2 T-cell effector functions and Vγ9Vδ2 T-cell expansion, six bispecific engagers were generated (see **Figure 2A** for design). The engagers included the bivalent Vδ2^hi-lo^ VHH either directly linked to a VHH directed against EGFR [clone 7D12 (42)], PSMA [clone JVZ-007 (43)] or CD1d [clone 1D12 (44)] at the N-terminus or (indirectly) linked to these VHHs using Fc domains that heterodimerize using knobs-into-holes technology (see Materials and Methods). The latter formats would allow for half-life extension of the engagers when applied *in vivo*.

All six constructs bound with similar EC_50_ values of 0.2-0.5 nM to Vγ9Vδ2 T-cells (**Figure 2B**) and supported significant Vγ9Vδ2 T-cell enrichment (median 20.6-58.6% of total T cells, versus 2.9% in IL-2 control) and expansion (median 12.2-32.4 fold expansion, versus 1.4 fold in IL-2 control) when added at a concentration of 1 nM (TAA-Vδ2^hi-lo^ bsVHH) or 100 nM (TAA-Vδ2^hi-lo^ bsVHH-Fc) to healthy donor derived PBMC for 8 days (optimal concentrations determined by titration, not shown), illustrating that the ability to induce Vγ9Vδ2 T-cell expansion was retained in these molecular formats (**Figure 2C**). Enrichment and expansion of Vγ9Vδ2 T-cells was not observed when a monovalent Vδ2-VHH was linked to the EGFR or PSMA-specific VHHs, underscoring the importance of including the bivalent Vδ2^hi-lo^ VHH when expansion is desired (**Supplementary Figures S3A, B**). Of note, enrichment and expansion of Vγ9Vδ2 T-cells was observed when PBMC were cultured with CD1d specific engagers containing either the monovalent Vδ2-VHH or the bivalent Vδ2^hi-lo^ VHH, which was likely related to CD1d expressing cells in PBMC (e.g. monocytes, B cells, circulating dendritic cells) that allowed Vδ2-TCR crosslinking to support Vγ9Vδ2 T-cell expansion (32).

After binding of these TAA-Vδ2^hi-lo^ bsVHH and TAA-Vδ2^hi-lo^ bsVHH-Fc molecules to the TAA expressing tumor cells was confirmed (**Figure 2D**), the antitumor effector functions of these molecules were evaluated in co-cultures of Vγ9Vδ2 T-cells and tumor cells expressing the respective TAAs (i.e. SW480 (EGFR^+^), 22Rv1 (PSMA^+^) or MM.1s.CD1d (CD1d^+^)). A representative gating strategy is shown in **Supplementary Figure S5A**. As shown in **Figures 2E, F**, all constructs were able to induce Vγ9Vδ2 T-cell degranulation and subsequent tumor cell lysis with EC_50s_ in the low-picomolar range. Addition of the Fc domain resulted in a 3-9 fold reduced potency for tumor lysis, possibly due to steric hindrance or an increased intermembrane distance between the Vγ9Vδ2 T-cell and TAA^+^ tumor target cell which may interfere with efficient immune synapse formation (46).

Incorporation of an anti-albumin binding domain can also provide a means to extend *in vivo* half-life (45). To explore this, we added an anti-albumin VHH to the EGFR-Vδ2^hi-lo^ bsVHH (see **Supplementary Figure S4A** for design) and confirmed binding to both Vγ9Vδ2 T-cells and EGFR^+^ tumor cells (**Supplementary Figure S4B**). The EGFR-Vδ2^hi-lo^ bsVHH-albumin retained the ability to support Vγ9Vδ2 T-cell enrichment and expansion in healthy donor PBMC (**Supplementary Figure S4C**) and also triggered Vγ9Vδ2 T-cell degranulation and lysis of SW480 tumor cells (**Supplementary Figure S4D**). The potency (i.e. EC_50_ for degranulation and tumor lysis) of the EGFR-Vδ2^hi-lo^ bsVHH-albumin construct was ~10 fold lower compared to the EGFR-Vδ2^hi-lo^ bsVHH-Fc perhaps as a result of impaired immune synapse formation when albumin was bound to the engager in this specific orientation.

### Vγ9Vδ2 T-cells expanded using TAA-Vδ2^hi-lo^ bsVHH and bsVHH-Fc are activated and perform cytotoxic effector functions when exposed to TAA expressing tumor cells

**Figure 3:**
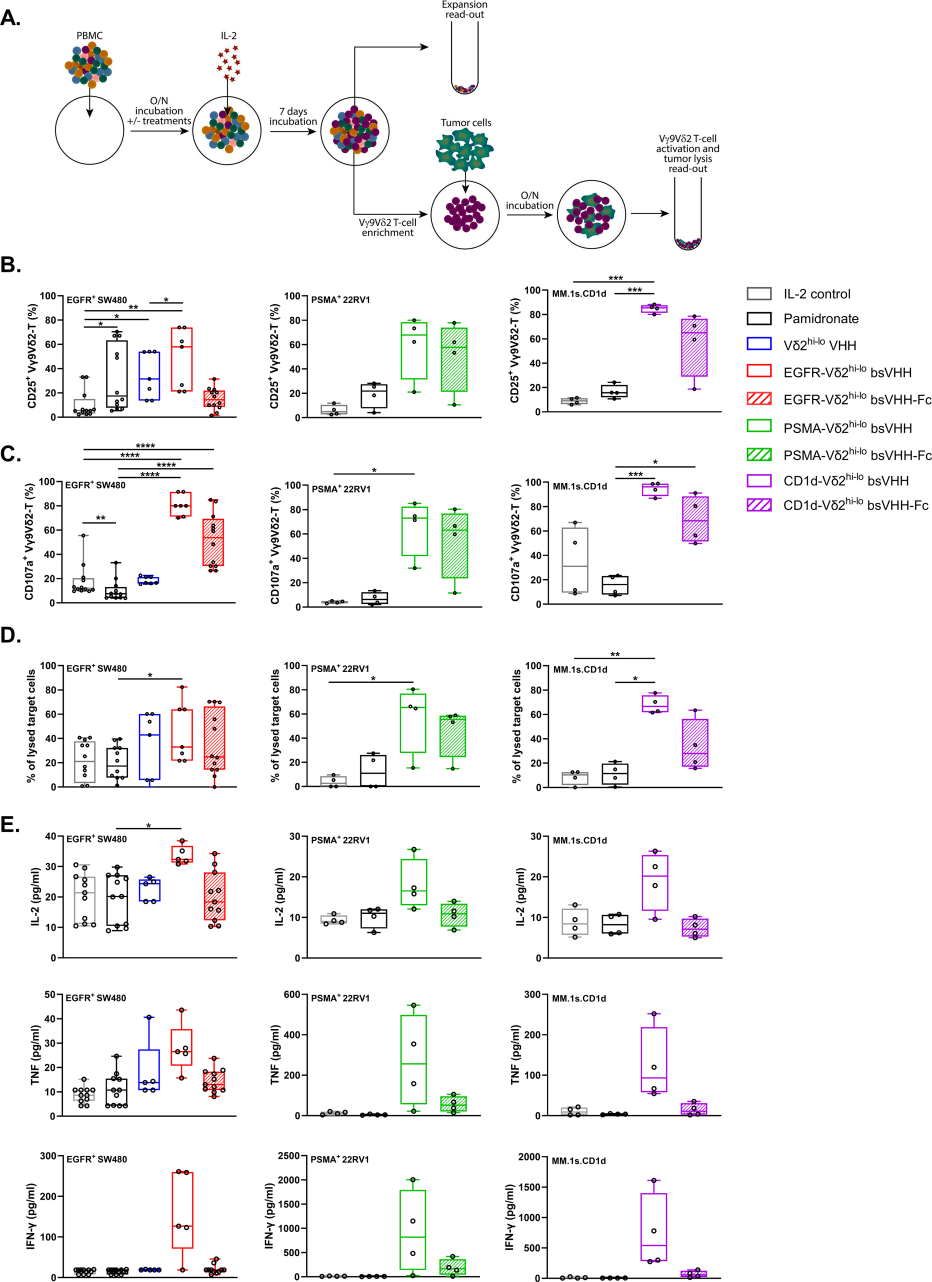
Vγ9Vδ2 T-cells expanded using TAA-Vδ2^hi-lo^ bsVHH and TAA-Vδ2^hi-lo^ bsVHH-Fc are activated and induce tumor lysis when exposed to TAA-expressing tumor cells. **(A)** Schematic overview of method used for PBMC culture, subsequent assessment of Vγ9Vδ2 T-cell expansion, as well as co-culture of enriched Vγ9Vδ2 T-cells and tumor cells. **(B–E)** Vγ9Vδ2 T-cell CD25 expression (**A**; n=4-12), CD107a expression (**B**; n=4-12), specific tumor cell lysis (**C**; n=4-12) and levels of IL-2, TNF and IFN-γ (pg/ml, **D**; n=4-5) in 24hr co-cultures of SW480 (EGFR^+^), 22Rv1 (PSMA^+^) or MM.1s.CD1d (CD1d^+^) tumor cells with Vγ9Vδ2 T-cells enriched (purity > 60%) from 8 day cultures of healthy donor PBMC in the presence or absence of 1 nM TAA-Vδ2^hi-lo^ bsVHH, 100 nM TAA-Vδ2^hi-lo^ bsVHH-Fc or 10 µM pamidronate (1:1 E:T ratio). Data generated through flow cytometry **(B–D)** or CBA **(E)**. Individual data-points are indicated using open circles and box and whisker plots indicate the median, 25th to 75th percentiles and minimum to maximum. One-way ANOVA with Tukey’s multiple comparisons test was used for statistical analysis; P=< 0.05: *, P=< 0.01: **, P=< 0.001: ***, P=< 0.0001: ****.

The functional properties of the expanded Vγ9Vδ2 T-cells were next explored. For this purpose, expanded Vγ9Vδ2 T-cells were enriched from the PBMC cultures using negative MACS isolation and co-cultured with either EGFR^+^ SW480 colorectal cancer cells, PSMA^+^ 22Rv1 prostate cancer cells, or CD1d^+^ MM.1s multiple myeloma cells for an additional 24 hr; the TAA-Vδ2^hi-lo^ bsVHH/bsVHH-Fc engagers were re-added to the respective conditions. **Figure 3A** illustrates the experimental design and **Supplementary Figures S5B, C** show the Vγ9Vδ2 T-cell frequency post expansion and post enrichment and a representative dot-plot thereof. Compared to Vγ9Vδ2 T-cells cultured with IL-2 only (IL-2 control), Vγ9Vδ2 T-cells expanded using pamidronate, the Vδ2^hi-lo^ bivalent VHH, or the Vδ2^hi-lo^ bsVHH/bsVHH-Fc typically expressed higher levels of the activation marker CD25 upon co-culture with tumor cells (**Figure 3B**). In the presence of the TAA-Vδ2^hi-lo^ bsVHH and bsVHH-Fc molecules, the expanded Vγ9Vδ2 T-cells expressed significantly higher levels of the degranulation marker CD107a and, in line, triggered more robust tumor lysis (**Figures 3C, D**; plots from a representative donor are shown in **Supplementary Figures S5D, E**). Supernatants obtained from these 24 hr co-cultures demonstrated that the TAA-Vδ2^hi-lo^ bsVHH molecules promoted the production of higher levels of various proinflammatory cytokines, i.e. IL-2, TNF and IFN-γ (**Figure 3E**). Overall, the increase in proinflammatory mediators and cytolytic effects were most pronounced when expanded Vγ9Vδ2 T-cells were cultured with TAA-Vδ2^hi-lo^ bsVHH. Taken together, Vγ9Vδ2 T-cells expanded from PBMC with the TAA-Vδ2^hi-lo^ bsVHH and bsVHH-Fc exhibit an activated effector phenotype and mediate tumor lysis when exposed to TAA expressing tumor cells.

### Vγ9Vδ2 T-cells can be expanded from cancer patient PBMC using TAA-Vδ2^hi-lo^ bsVHH and bsVHH-Fc

**Figure 4:**
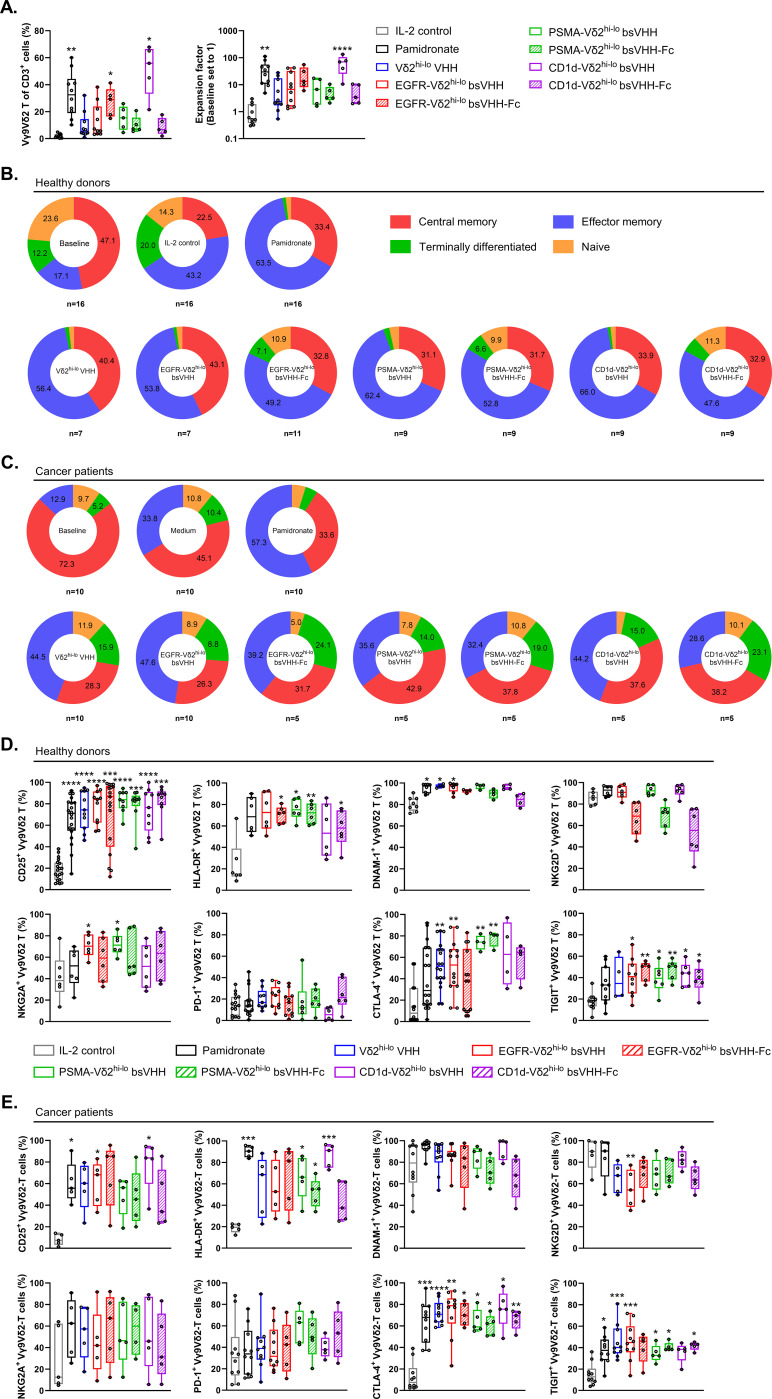
Bivalent Vδ2^hi-lo^ VHH, TAA-Vδ2^hi-lo^ bsVHH and TAA-Vδ2^hi-lo^ bsVHH-Fc support Vγ9Vδ2 T-cell expansion in cancer patient PBMC and expanded Vγ9Vδ2 T-cells display a memory dominated phenotype. **(A)** Enrichment (left panel) and fold expansion (right panel) of Vγ9Vδ2 T-cells during an 8 day culture of cancer patient PBMC in the presence or absence of 1nM bivalent Vδ2^hi-lo^ VHH, 1 nM TAA-Vδ2^hi-lo^ bsVHH, 100nM TAA-Vδ2^hi-lo^ bsVHH-Fc or 10 µM pamidronate (n=5-10). **(B, C)** Proportion (%) of central memory (CD27^+^ CD45RA^-^), effector memory (CD27^-^ CD45RA^-^), terminally differentiated (CD27^-^ CD45RA^+^) or naïve (CD27^+^ CD45RA^+^) cells within total Vγ9Vδ2 T-cell population before (baseline) and after an 8 day culture in the presence or absence of 1 nM TAA-Vδ2^hi-lo^ bsVHH, 100nM TAA-Vδ2^hi-lo^ bsVHH-Fc or 10 µM pamidronate (n=5-16) using healthy donor PBMC **(B)** or cancer patient PBMC **(C)**. **(D, E)** Expression of CD25, HLA-DR, DNAM-1, NKG2D, NKG2A, PD-1, CTLA-4 and TIGIT on Vγ9Vδ2 T-cells expanded for 8 days in the presence or absence of 1 nM TAA-Vδ2^hi-lo^ bsVHH, 100nM TAA-Vδ2^hi-lo^ bsVHH-Fc or 10 µM pamidronate (data reflect % positive of total Vγ9Vδ2 T-cell fraction; n=5-17) using healthy donor PBMC **(D)** or cancer patient PBMC **(E)**. Data generated using flow cytometry. Individual data-points are indicated using open circles and box and whisker plots indicate the median, 25th to 75th percentiles and minimum to maximum **(A, D, E)**. One-way ANOVA with Tukey’s multiple comparisons test was used for statistical analysis and asterisks are shown compared to IL-2 control; P=< 0.05: *, P=< 0.01: **, P=< 0.001: ***, P=< 0.0001: ****.

We next evaluated whether the TAA-Vδ2^hi-lo^ bsVHH and bsVHH-Fc molecules could also support expansion of Vγ9Vδ2 T-cells from cancer patient PBMC. For this purpose, PBMC were obtained from patients with gastric cancer, esophageal cancer or melanoma. The majority of these patients had advanced-stage disease and was treatment naïve (see **Supplementary Table S3** for patient characteristics). As shown in **Figure 4A**, pamidronate, the bivalent Vδ2^hi-lo^ VHH as well as the TAA-Vδ2^hi-lo^ bsVHH and TAA-Vδ2^hi-lo^ bsVHH-Fc constructs supported the enrichment and expansion of Vγ9Vδ2 T-cells in 8 day PBMC cultures. Enrichment (55.9 ± 33.2%; median ± IQR) and expansion (69.8 ± 79.5 fold; median ± IQR) was most pronounced with the CD1d-Vδ2^hi-lo^ bsVHH and less striking when the Fc domain was connected to this bsVHH. With the EGFR and PSMA specific Vδ2^hi-lo^ engagers, expansion was similar in the presence and absence of the Fc domain.

### TAA-Vδ2^hi-lo^ bsVHH and bsVHH-Fc expanded Vγ9Vδ2 T-cells display a central/effector memory phenotype in healthy donors and retain a diverse memory dominated phenotype in cancer patients

The phenotype of Vγ9Vδ2 T-cells expanded from healthy donor and cancer patient PBMC using pamidronate, the Vδ2^hi-lo^ bivalent VHH, the various TAA-Vδ2^hi-lo^ bsVHH and TAA-Vδ2^hi-lo^ bsVHH-Fc molecules and the IL-2 control was characterized (representative gating strategy shown in **Supplementary Figure S6**). At baseline, most Vγ9Vδ2 T-cells in healthy donor PBMC had a central memory (CD27^+^CD45RA^-^, 47.1 ± 4.5%; mean ± SEM) phenotype with naïve (CD27^+^CD45RA^+^, 23.6 ± 4.2%; mean ± SEM), effector memory (CD27^-^ CD45RA^-^, 17.1 ± 4.6%; mean ± SEM) and terminally differentiated (CD27^-^CD45RA^+^, 12.1 ± 1.9%; mean ± SEM) Vγ9Vδ2 T-cell populations representing smaller fractions (**Figure 4B**). After an 8-day culture of PBMC with 100 IU/ml IL-2, pamidronate or the various TAA-Vδ2^hi-lo^ bsVHH and bsVHH-Fc engagers, expanded Vγ9Vδ2 T-cells exhibited a shift towards an effector memory phenotype with a concomitant reduction in the frequency of naïve and terminally differentiated subsets while the fraction of central memory cells overall remained similar in size. Of interest, Vγ9Vδ2 T-cells expanded with the TAA-Vδ2^hi-lo^ bsVHH-Fc molecules retained larger proportions of Vγ9Vδ2 T-cells with a terminally differentiated phenotype (EGFR-Vδ2^hi-lo^ bsVHH; *P* = 0.0007, PSMA-Vδ2^hi-lo^ bsVHH; *P* = 0.02, CD1d-Vδ2^hi-lo^ bsVHH; *P* = 0.01, unpaired *t* tests) and naïve phenotype (EGFR-Vδ2^hi-lo^ bsVHH; *P* = 0.008, PSMA-Vδ2^hi-lo^ bsVHH; *P* = 0.0002, CD1d-Vδ2^hi-lo^ bsVHH; *P* = 0.002, unpaired *t* tests). When compared to Vγ9Vδ2 T-cells in healthy donor PBMC, the proportion of central memory Vγ9Vδ2 T-cells was increased in cancer patient PBMC at baseline (72.2 ± 6.8% versus 47.1 ± 4.4% in healthy donors; mean ± SEM, *P* = 0.001; unpaired *t* test). This was accompanied by a lower proportion of Vγ9Vδ2 T-cells with a naïve phenotype (9.7 ± 2.6% versus 23.6 ± 4.2% in healthy donors; mean ± SEM, *P* = 0.02; unpaired *t* test), which may suggest their priming and involvement in the antitumor immune response (**Figure 4C**). The Vγ9Vδ2 T-cells expanded from cancer patient PBMC using the TAA-Vδ2^hi-lo^ bsVHH and bsVHH-Fc were in general skewed to an effector memory phenotype. However, while the proportion of naïve and terminally differentiated effector memory cells was strongly reduced upon expansion in Vγ9Vδ2 T-cells expanded from healthy donor PBMC, these were maintained in Vγ9Vδ2 T-cells expanded from cancer patient PBMC (**Figure 4C**).

Healthy donor Vγ9Vδ2 T-cells expanded by the TAA-Vδ2^hi-lo^ bsVHH and bsVHH-Fc constructs as well as pamidronate were highly activated as shown by strong increases in CD25 and HLA-DR expression (**Figure 4D**). During culture, expression of DNAM-1, an activating receptor expressed by most Vγ9Vδ2 T-cells, remained highly expressed and was even expressed by a larger proportion in several cases. Of interest, NKG2D, an activating receptor also expressed by most Vγ9Vδ2 T-cells, remained highly expressed when Vγ9Vδ2 T-cells were expanded with pamidronate or the TAA-Vδ2^hi-lo^ bsVHH, but was expressed by a lower proportion of Vγ9Vδ2 T-cells when these were expanded with the TAA-Vδ2^hi-lo^ bsVHH-Fc engagers, though this did not reach statistical significance. The upregulation of activation markers on expanded Vγ9Vδ2 T-cells was accompanied by an increase in the proportion of Vγ9Vδ2 T-cells expressing co-inhibitory receptors including NKG2A, CTLA-4, and TIGIT. Of note, the proportion of Vγ9Vδ2 T-cells expressing the inhibitory immune checkpoint receptor PD-1 was not altered. Overall, the increased expression of multiple inhibitory receptors on expanded Vγ9Vδ2 T-cells likely reflects their increased activation state (47, 48). Indeed, NKG2A was previously reported to identify a population of Vγ9Vδ2 T-cells with greater cytotoxic potential (11). In the conditions where cancer patient PBMC were cultured with pamidronate or the Vδ2^hi-lo^ bsVHH containing constructs, Vγ9Vδ2 T-cells expressed increased levels of the activation markers CD25 and HLA-DR (**Figure 4E**). At baseline, expression of DNAM-1 and NKG2D was more variable on cancer patient Vγ9Vδ2 T-cells and expression did not significantly change upon expansion with the TAA-Vδ2^hi-lo^ bsVHH and bsVHH-Fc molecules. Of interest, while PD-1 was typically expressed by a low proportion of Vγ9Vδ2 T-cells in healthy donor PBMC at baseline, more variable expression was noted on Vγ9Vδ2 T-cells in cancer patient PBMC with a further increase in the proportion of PD-1 expressing Vγ9Vδ2 T-cells noted in several patients after expansion with either pamidronate or the Vδ2^hi-lo^ bsVHH constructs. NKG2A expression by Vγ9Vδ2 T-cells was variable at both baseline and upon expansion. CTLA-4 and TIGIT were expressed by an approximately similar proportion of Vγ9Vδ2 T-cells in cancer patient and healthy donor PBMC and were in both cases upregulated upon expansion, though the proportion of Vγ9Vδ2 T-cells expressing CTLA-4 appeared to be more consistently increased upon activation and expansion in cancer patient PBMC.

### CD1d-Vδ2^hi-lo^ bsVHH construct induces expansion of Vγ9Vδ2 T-cells *in vivo*

**Figure 5:**
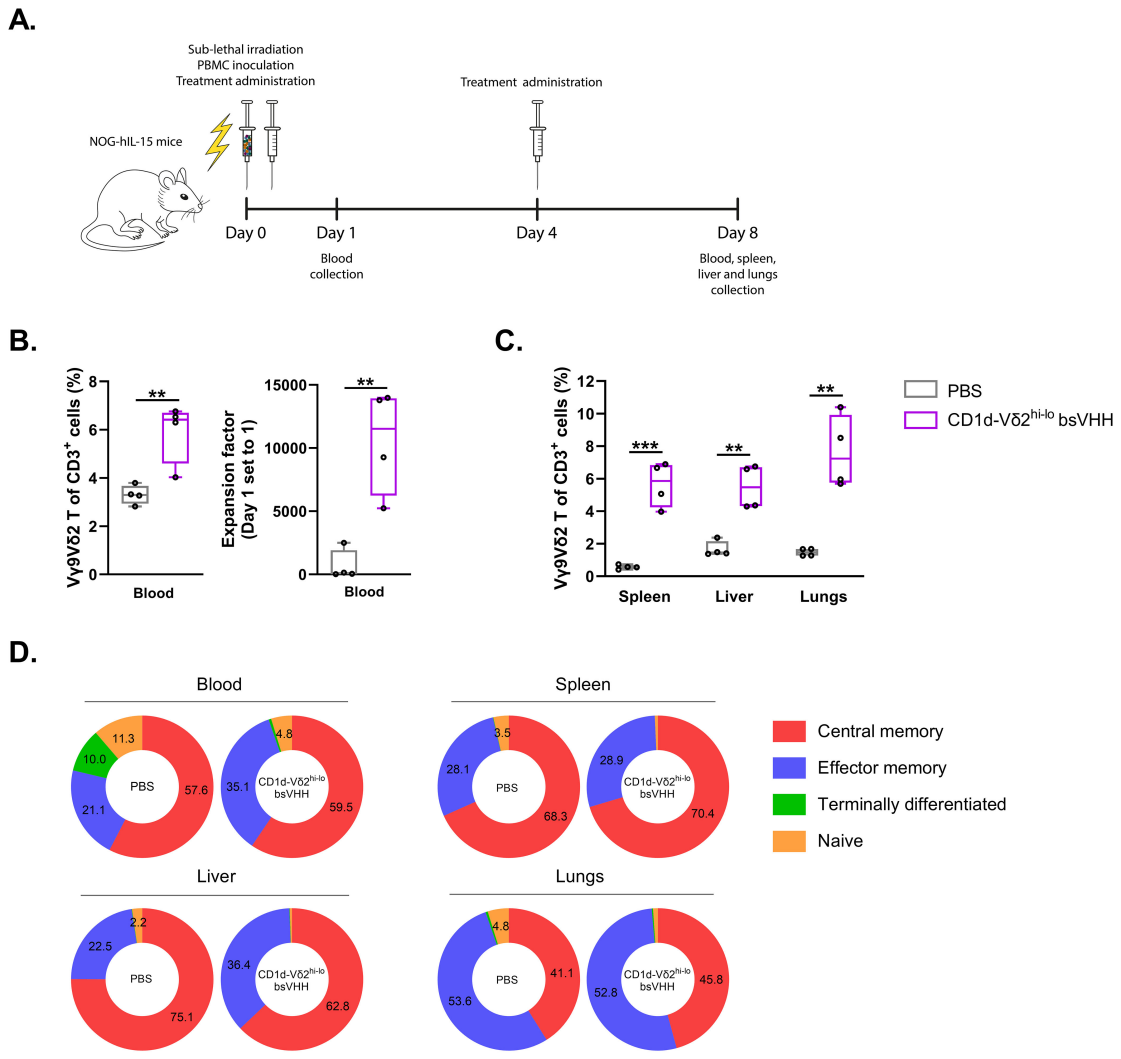
CD1d-Vδ2^hi-lo^ bsVHH induces Vγ9Vδ2 T-cell expansion in NOG-hIL-15 mice. NOG-hIL-15 mice were sub-lethally irradiated and i.v. inoculated with human PBMC on day 0 and treated i.p. with PBS (control group) or 0.5 mg/kg CD1d-Vδ2^hi-lo^ bsVHH on day 0 and 4 (n=4 mice per group). **(A)** Timeline of the *in vivo* study. **(B)** Enrichment (left panel) and fold expansion (right panel) of human PBMC-derived Vγ9Vδ2 T-cells *in vivo* 8 days after PBMC inoculation and two doses of i.p. injection with PBS or CD1d-Vδ2^hi-lo^ bsVHH. **(C)** Enrichment of human PBMC-derived Vγ9Vδ2 T-cells in spleen, liver and lungs 8 days after PBMC inoculation and two doses of i.p. injection with PBS or CD1d-Vδ2^hi-lo^ bsVHH. **(D)** Phenotype of expanded human PBMC-derived Vγ9Vδ2 T-cells *in vivo* from blood, spleen, liver and lungs 8 days after PBMC inoculation and two doses of i.p. injection with PBS or CD1d-Vδ2^hi-lo^ bsVHH. Data generated using flow cytometry. Individual data-points are indicated using open circles and box and whisker plots indicate the median, 25th to 75th percentiles and minimum to maximum. Unpaired *t* tests were used for statistical analysis; P=< 0.01: **, P=< 0.001: ***.

While all TAA-Vδ2^hi-lo^ bsVHHs supported expansion of Vγ9Vδ2 T-cells, results were consistently robust with the CD1d-Vδ2^hi-lo^ bsVHH and this engager was therefore selected to assess whether Vγ9Vδ2 T-cell expansion could also be induced *in vivo*. As mice lack the phosphoantigen responsive γδ T-cell population that humans have, an immunodeficient NOG-hIL-15 mouse model was selected as this allowed for inoculation of human PBMC and could simultaneously provide a relatively low level of IL-15 cytokine support. Mice (n=4/group) were i.v. inoculated with human PBMC (n=1 healthy donor with 0.89% Vγ9Vδ2 T-cell of total CD3^+^ T-cells) on day 0 followed by i.p. administration of either PBS or CD1d-Vδ2^hi-lo^ bsVHH on days 0 and 4 (**Figure 5A**). On day 8, animals were sacrificed and the proportion of Vγ9Vδ2 T-cells was assessed in peripheral blood, spleen, liver and lungs (gating strategy illustrated in **Supplementary Figure S7**). As shown in **Figures 5B, C**, the proportion of Vγ9Vδ2 T-cells was significantly higher in mice treated with the CD1d-Vδ2^hi-lo^ bsVHH compared to the PBS control. Administration of the CD1d-Vδ2^hi-lo^ bsVHH resulted in an impressive 11525 (± 7692; median ± IQR, *P* = 0.004) fold expansion of the Vγ9Vδ2 T-cells in peripheral blood. CD1d-Vδ2^hi-lo^ bsVHH expanded Vγ9Vδ2 T-cells isolated from spleen, liver and lungs all expressed a central or effector memory phenotype, whereas 4.8% (± 1.0; mean ± SEM) of the Vγ9Vδ2 T-cells in peripheral blood retained a naïve phenotype (**Figure 5D**). In summary, these results indicate that Vδ2^hi-lo^ bsVHH based engagers can also trigger expansion of Vγ9Vδ2 T-cells in a NOG-hIL-15 mouse *in vivo* model.

## Discussion

Vγ9Vδ2 T-cells are a relatively homogeneous population of antitumor immune effector cells capable of inducing cytotoxicity in a wide range of malignancies independently of HLA, which makes them highly promising for cancer immunotherapy (8). Early clinical trials that attempted to exploit the therapeutic potential of Vγ9Vδ2 T-cells were based on activating and expanding Vγ9Vδ2 T-cells either *ex vivo*, followed by ACT, or directly *in vivo* using N-BP or BrHPP alone or in combination with IL-2. These approaches were shown to be safe and tolerable (49–62), although clinical results lacked consistency (49, 51, 52, 59, 62, 63), which may have been related to the absence of a specific tumor targeting moiety, blunting of Vγ9Vδ2 T-cell responsiveness with repeated administration of N-BP/BrHPP and the donor-to-donor variability in the size of the Vγ9Vδ2 T-cell population (49, 51, 54, 56). Recently, strategies that drive tumor selective activation of Vγ9Vδ2 T-cells, e.g. using CARs or bispecific antibodies, have emerged as promising novel therapeutic approaches (8). While production of CAR (Vγ9Vδ2) T-cells is considered laborious and costly, and requires preconditioning lymphodepleting chemotherapy regimens before administration to patients, bispecific antibodies could provide a more straightforward *off-the-shelf* approach. We and others have recently demonstrated the potential of bispecific Vγ9Vδ2 T-cell engagers to elicit potent tumor cell lysis (31–37, 64, 65). As these engagers rely on the endogenous Vγ9Vδ2 T-cell population, which, as this study confirms, varies between individuals and with age (9–14), and do not consistently induce Vγ9Vδ2 T-cell expansion, we evaluated whether a bispecific antibody that is bivalent, rather than monovalent, for Vδ2-TCR binding, could combine tumor specific activation with consistent expansion of Vγ9Vδ2 T-cells. Our data show that a bivalent VHH that links a high and low affinity Vδ2-TCR specific VHH can support Vγ9Vδ2 T-cell expansion *in vitro*, *ex vivo* and *in vivo* and that this ability is maintained when incorporated in bispecific formats, allowing lysis of TAA expressing tumor cells, also in combination with an Fc domain or anti-albumin binding unit for plasma half-life extension.

From an available panel of Vδ2-TCR specific VHHs, three were selected based on low (VHH-5C7), intermediate (VHH-5D3) and high (VHH-6H4) affinity (41) and linked in different combinations and orientations. Only bivalent VHHs that combined a high and low affinity Vδ2-TCR specific VHH supported consistent and potent Vγ9Vδ2 T-cell enrichment and expansion, particularly with N-terminal positioning of the high affinity Vδ2-VHH and independent of the explored linker lengths. As Vγ9Vδ2 T-cells are potent effector cells, one can envision that the bivalent Vδ2-VHHs containing combinations of high and intermediate affinity Vδ2-VHHs resulted in clustering of activated Vγ9Vδ2 T-cells and limited subsequent expansion through fratricide or activation-induced cell death due to too strong TCR triggering.

When the bivalent Vδ2^hi-lo^ VHHs were linked to VHHs directed against a variety of TAA specific VHHs (i.e. EGFR, PSMA or CD1d, molecules that can be over-expressed by tumor cells (31, 32, 66)) alone or with an Fc or anti-albumin binding domain for half-life extension, enrichment and expansion were maintained at levels similar to those obtained with the N-BP pamidronate. In contrast to N-BP expanded Vγ9Vδ2 T-cells, TAA-Vδ2^hi-lo^ VHH and bsVHH-Fc expanded Vγ9Vδ2 T-cells had the additional ability to specifically engage and lyse TAA expressing tumor cells. Importantly, the TAA-Vδ2^hi-lo^ bsVHH and bsVHH-Fc also supported Vγ9Vδ2 T-cell expansion using PBMC from patients with gastric cancer, esophageal cancer and melanoma. At baseline, cancer patient Vγ9Vδ2 T-cells contained a higher proportion of central memory and a lower proportion of naïve Vγ9Vδ2 T-cells compared to healthy individuals, which may reflect a natural interaction of Vγ9Vδ2 T-cells with tumor cells and differentiation induction through pAg/BTNs. PD-1 was typically expressed by a low proportion of Vγ9Vδ2 T-cells in healthy individuals but was more variable among cancer patients. Upon expansion by TAA-Vδ2^hi-lo^ bsVHH and bsVHH-Fc, activation markers CD25 and HLA-DR were more frequently expressed as were inhibitory receptors CTLA-4 and TIGIT. In healthy donor PBMC, this was accompanied by an increase in the proportion of NKG2A expressing Vγ9Vδ2 T-cells, while the fraction of Vγ9Vδ2 T-cells expressing PD-1 did not change. In cancer patient PBMC, expression of PD-1 and NKG2A did not statistically significantly change on Vγ9Vδ2 T-cells upon expansion. The proportion of Vγ9Vδ2 T-cells expressing activating receptors NKG2D and DNAM-1 remained relatively high, and this in combination with an increase in the expression of activation markers and several inhibitory receptors likely reflects an overall cytotoxic phenotype of the expanded Vγ9Vδ2 T-cells. Indeed, NKG2A^+^ Vγ9Vδ2 T-cells were found to be highly cytolytic (11). In line, we demonstrated that Vγ9Vδ2 T-cells expanded with the TAA-Vδ2^hi-lo^ bsVHH and bsVHH-Fc molecules retained the potential to degranulate, produce proinflammatory cytokines and lyse tumor cells in a TAA specific fashion. Combining immune checkpoint inhibitors with TAA-Vδ2^hi-lo^ bsVHH or bsVHH-Fc molecules could be of potential interest, as this strategy may further enhance the antitumor response of the engaged Vγ9Vδ2 T-cells.

The addition of a half-life extension domain to the TAA-Vδ2^hi-lo^ bsVHH did typically impact the Vγ9Vδ2 T-cell expansion, phenotypic alterations and/or effector functions. This may be due to steric hindrance or an increased intermembrane distance between the Vγ9Vδ2 T-cell and TAA^+^ tumor target cell interfering with efficient immune synapse formation (46) as a result of either the Fc domain or a bound albumin molecule, translating into higher EC_50s_ for degranulation and tumor lysis compared to the (non-Fc, non-anti-albumin VHH bound) TAA-Vδ2^hi-lo^ bsVHH.

To explore the ability of the bivalent Vδ2^hi-lo^ VHH based approach to similarly support expansion of Vγ9Vδ2 T-cells *in vivo*, one of the TAA-Vδ2^hi-lo^ bsVHH was also administered to NOG-hIL-15 mice inoculated with human PBMC. Robust expansion of Vγ9Vδ2 T-cells with a predominant central and effector memory phenotype was observed in peripheral blood as well as in spleen, liver and lungs. Notably, although a small fraction of the TAA-Vδ2^hi-lo^ bsVHH expanded Vγ9Vδ2 T-cells in peripheral blood retained a naïve phenotype, no naïve Vγ9Vδ2 T-cells were observed in the spleen, liver or lungs likely indicating that only Vγ9Vδ2 T-cells with a central memory and effector memory phenotype infiltrated these organs after expansion.

Overall, our results demonstrate that bispecific Vδ2 T-cell engagers that combine a tumor (TAA) specific VHH and a Vδ2^hi-lo^ bivalent VHH can uniquely trigger both TAA specific lysis and support expansion of Vγ9Vδ2 T-cells. As Vγ9Vδ2 T-cell frequencies are variable and may be impacted by the generally advanced age of patients with cancer and by cancer related therapies (67), the here reported approach may promote the antitumor activity of bispecific Vγ9Vδ2 T-cell engagers by increasing the number of functional effector Vγ9Vδ2 T-cells available for tumor lysis and warrants further exploration in the context of both solid and hematologic malignancies.

## Data Availability

The original contributions presented in the study are included in the article/**Supplementary Material**, further inquiries can be directed to the corresponding author.
