# Supplementary material for: Vδ2 T-cell engagers bivalent for Vδ2-TCR binding provide anti-tumor immunity and support robust Vγ9Vδ2 T-cell expansion
Source: Front Oncol. 2024 Oct 18;14:1474007. doi: 10.3389/fonc.2024.1474007 (PMC11527600; doi:10.3389/fonc.2024.1474007)
Supplement: Supplementary file 1 [file DataSheet1.docx]

**
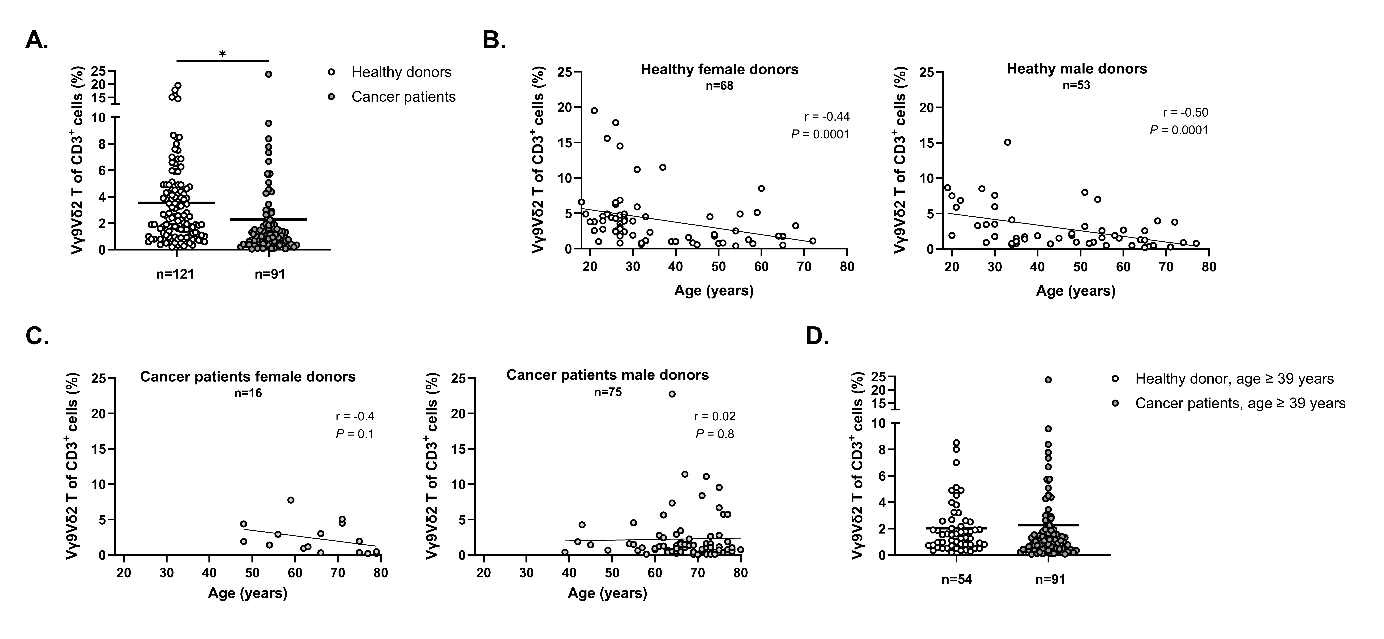
Figure S1.**

Vγ9Vδ2 T-cell frequency in healthy donors and cancer patients and correlation with donor age. **(A)** Vγ9Vδ2 T-cell frequency (percentage of total T cells) in healthy donor (n=121) and cancer patient (n=91) PBMC (horizontal line reflects the mean, *P* = 0.01). **(B)** Correlation between Vγ9Vδ2 T-cell frequency (percentage of total T cells) and age in healthy female (left panel, n=68; r = -0.44, *P* = 0.0001) and healthy male (right panel, n=53; r = -0.50, *P* = 0.0001) donors. **(C)** Correlation between Vγ9Vδ2 T-cell frequency (percentage of total T cells) and age in female cancer patients (left panel, n=16; r = -0.4, *P* = 0.1) and male cancer patients (right panel, n=75; r = 0.02, *P* = 0.8). **(D)** Vγ9Vδ2 T-cell frequency (percentage of total T cells) in PBMC of healthy donors (n=54) and cancer patients (n=91) ≥ 39 yr (horizontal line reflects the mean, *P* = 0.6). Data generated using flow cytometry. Unpaired *t* test (A, D) and Spearman’s rank correlations analysis (B, C) were used for statistical analysis. Individual data-points are indicated using open circles.

**
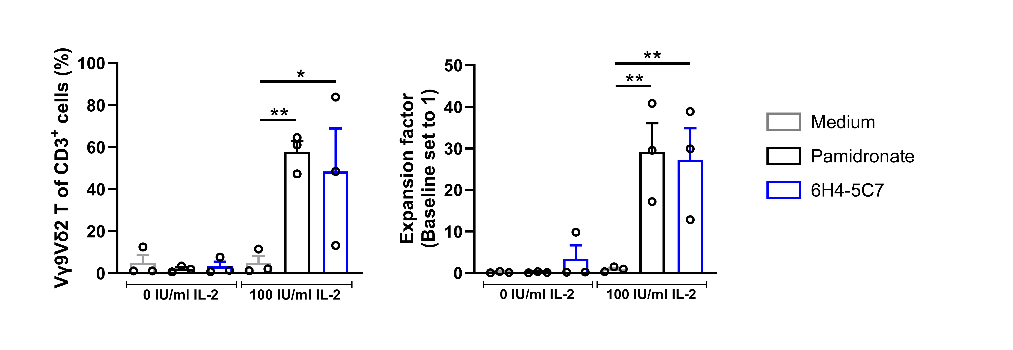
Figure S2.**

Effect of IL-2 on the 6H4-5C7 bivalent VHH and pamidronate induced enrichment and expansion of Vγ9Vδ2 T-cells. Enrichment (left panel) and fold expansion (right panel) of Vγ9Vδ2 T-cells during an 8 day culture of healthy donor PBMC (n=3) in the presence or absence of 1 nM 6H4-5C7 bivalent Vδ2-VHH or 10 µM pamidronate ± 100 IU/ml IL-2. Data generated using flow cytometry. Individual data-points are indicated using open circles, and bars indicate mean and SEM. Two-way ANOVA with Tukey’s multiple comparisons test was used for statistical analysis; P=< 0.05: *, P=< 0.01: **.


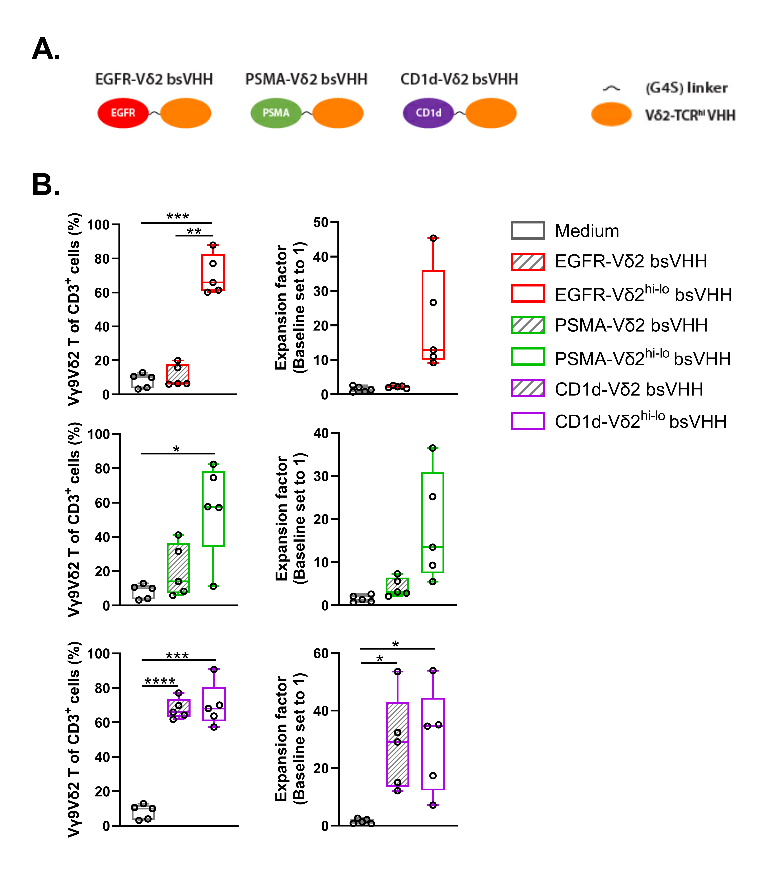
**Figure S3.**

Comparison of TAA-Vδ2 bsVHH and TAA-Vδ2^hi-lo^ bsVHH for induction of Vγ9Vδ2 T-cell enrichment and expansion. **(A)** Illustration of bispecific engagers monovalent for Vδ2 and TAA specific VHHs. **(B)** Enrichment (left panel) and fold expansion (right panel) of Vγ9Vδ2 T-cells during an 8 day culture of healthy donor PBMC in the presence or absence of 1 nM TAA-Vδ2 bsVHH or TAA-Vδ2^hi-lo^ bsVHH (n=5). Data generated using flow cytometry. Individual data-points are indicated using open circles and box and whisker plots indicate the median, 25th to 75th percentiles and minimum to maximum. One-way ANOVA with Tukey’s multiple comparisons test was used for statistical analysis; P=< 0.05: *, P=< 0.01: **, P=< 0.001: ***, P=< 0.0001: ****.

***
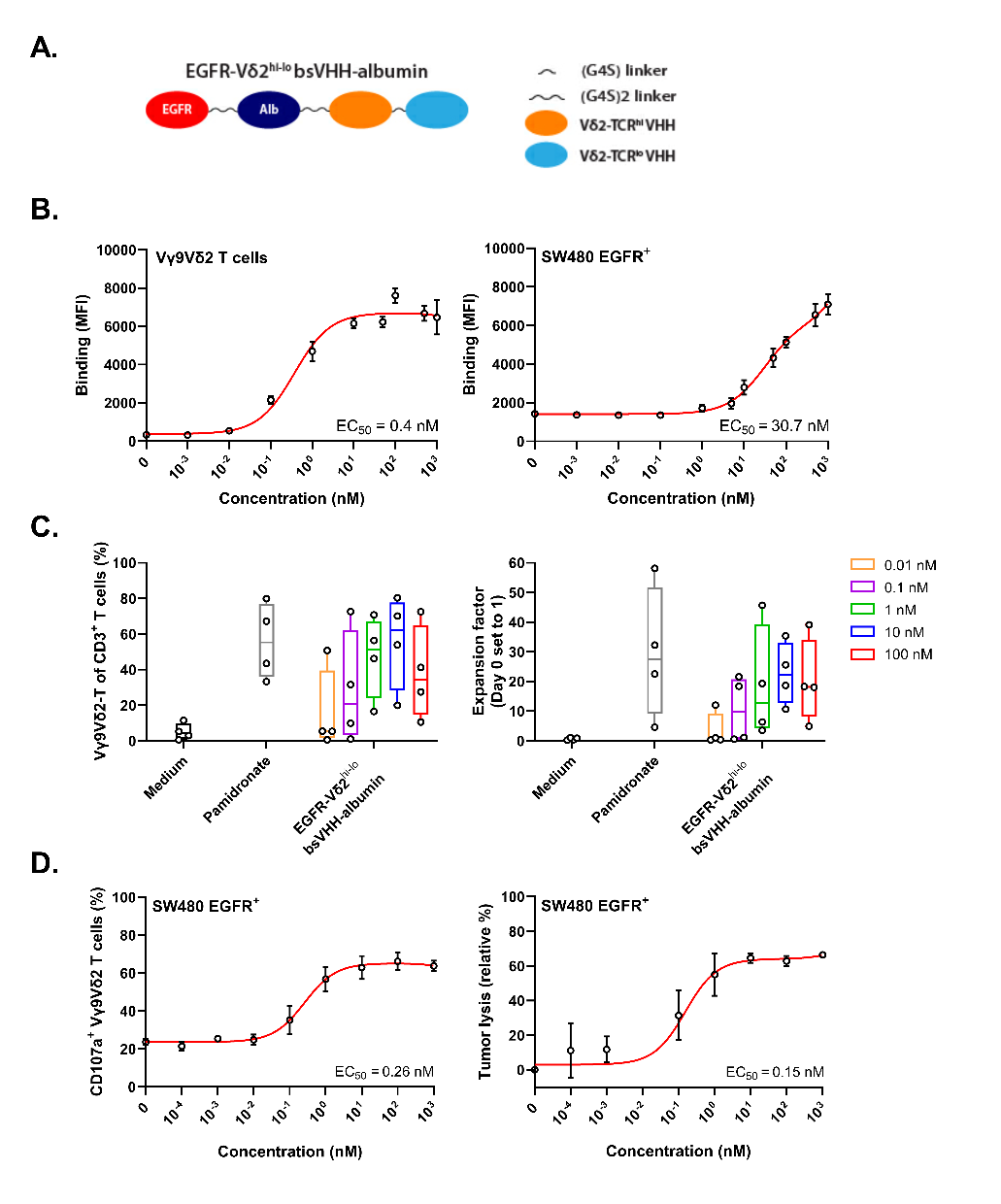
*Figure S4.**

EGFR-Vδ2^hi-lo^ bsVHH with an anti-albumin VHH support Vγ9Vδ2 T-cell expansion and trigger degranulation and tumor cell lysis. **(A)** Illustration of EGFR-Vδ2^hi-lo^ bsVHH-albumin. **(B)** Binding of the EGFR-Vδ2^hi-lo^ bsVHH-albumin to Vγ9Vδ2 T-cells and SW480 (EGFR^+^) tumor cells. Data represent mean and SEM (n=4). **(C)** Enrichment (left panel) and fold expansion (right panel) of Vγ9Vδ2 T-cells during an 8 day culture of healthy donor PBMC ± concentration range of EGFR-Vδ2^hi-lo^ bsVHH-albumin or 10 µM pamidronate. Individual data-points are indicated using open circles and box and whisker plots indicate the median, 25th to 75th percentiles and minimum to maximum (n=4). **(D)** Vγ9Vδ2 T-cell CD107a expression and specific lysis of SW480 cells after a 24 hr co-culture of Vγ9Vδ2 T-cells and SW480 tumor cells (1:1 E:T ratio) ± concentration range of EGFR-Vδ2^hi-lo^ bsVHH-abumin. Data represent mean and SEM (n=3). All data generated through flow cytometry. Two-way ANOVA with Tukey’s multiple comparisons test was used for statistical analysis (C).


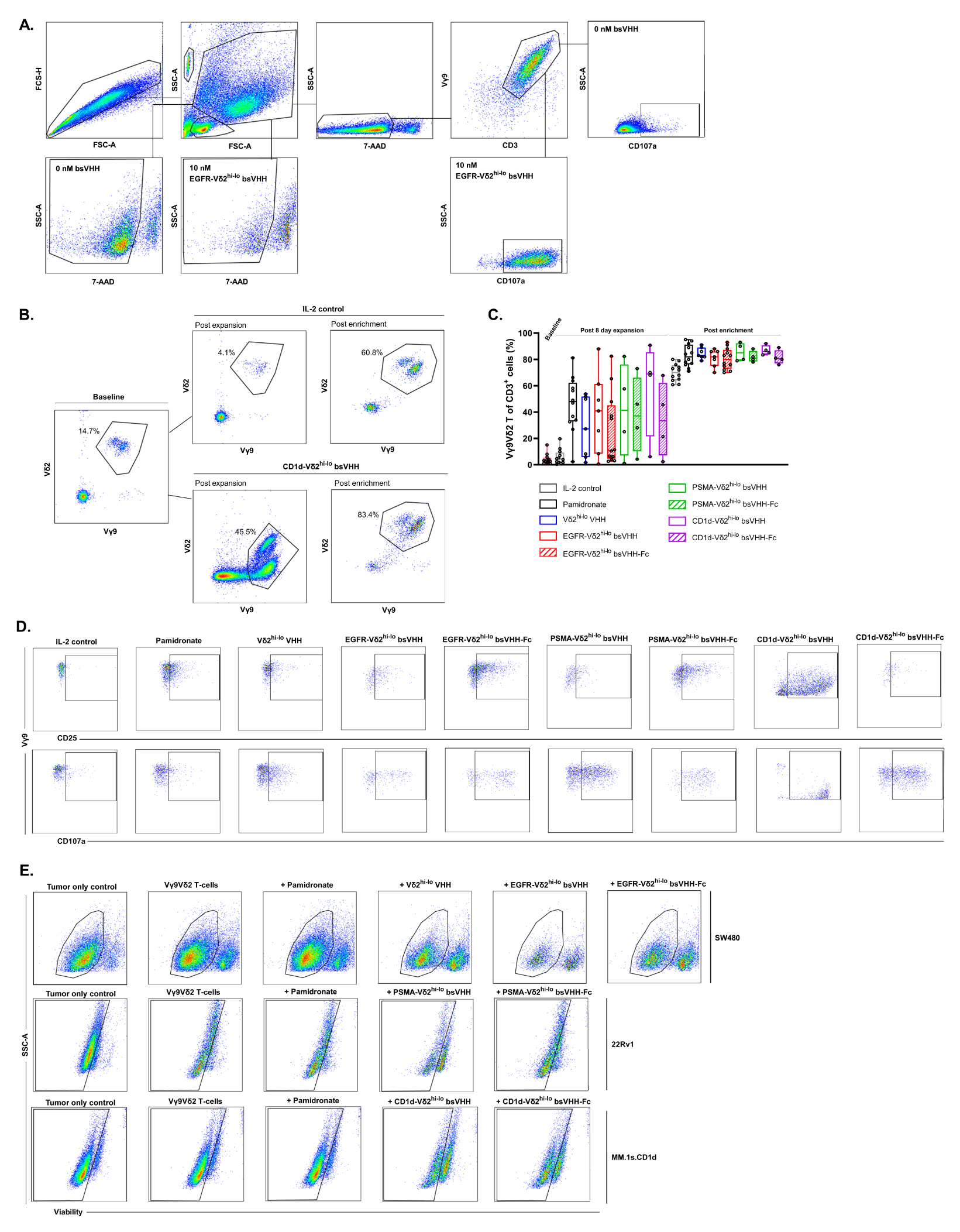


**Figure S5.** (**A**) Gating strategy used to analyze CD107a^+^ Vγ9Vδ2 T-cell frequencies and tumor cytotoxicity. Plots are derived from 24 hr co-cultures of Vγ9Vδ2 T-cells and SW480 tumor cells (1:1 E:T ratio) ± 10 nM EGFR-Vδ2^hi-lo^ bsVHH. (**B-C**) Vγ9Vδ2 T-cell frequencies at baseline, post expansion (day 8) and post enrichment (negative MACS isolation), (**B**) Dot-plots from a representative donor and (**C**) combined data of n=4-12 donors. (**D**) Plots from a representative donor are shown of Vγ9Vδ2 T-cell CD25 expression and CD107a expression in 24hr co-cultures of tumor cells with Vγ9Vδ2 T-cells enriched (purity > 60%) from 8 day cultures of healthy donor PBMC in the presence or absence of 1 nM TAA-Vδ2^hi-lo^ bsVHH, 100 nM TAA-Vδ2^hi-lo^ bsVHH-Fc or 10 µM pamidronate (1:1 E:T ratio). Plots for IL-2 and pamidronate controls are representative across different tumor cell lines used (shown here are SW480 cultures). (**E**) Plots from a representative donor are shown of tumor cytotoxicity in 24h co-cultures with Vγ9Vδ2 T-cells enriched (purity > 60%) from 8 day cultures of healthy donor PBMC in the presence or absence of 1 nM TAA-Vδ2^hi-lo^ bsVHH, 100 nM TAA-Vδ2^hi-lo^ bsVHH-Fc or 10 µM pamidronate (1:1 E:T ratio).

**
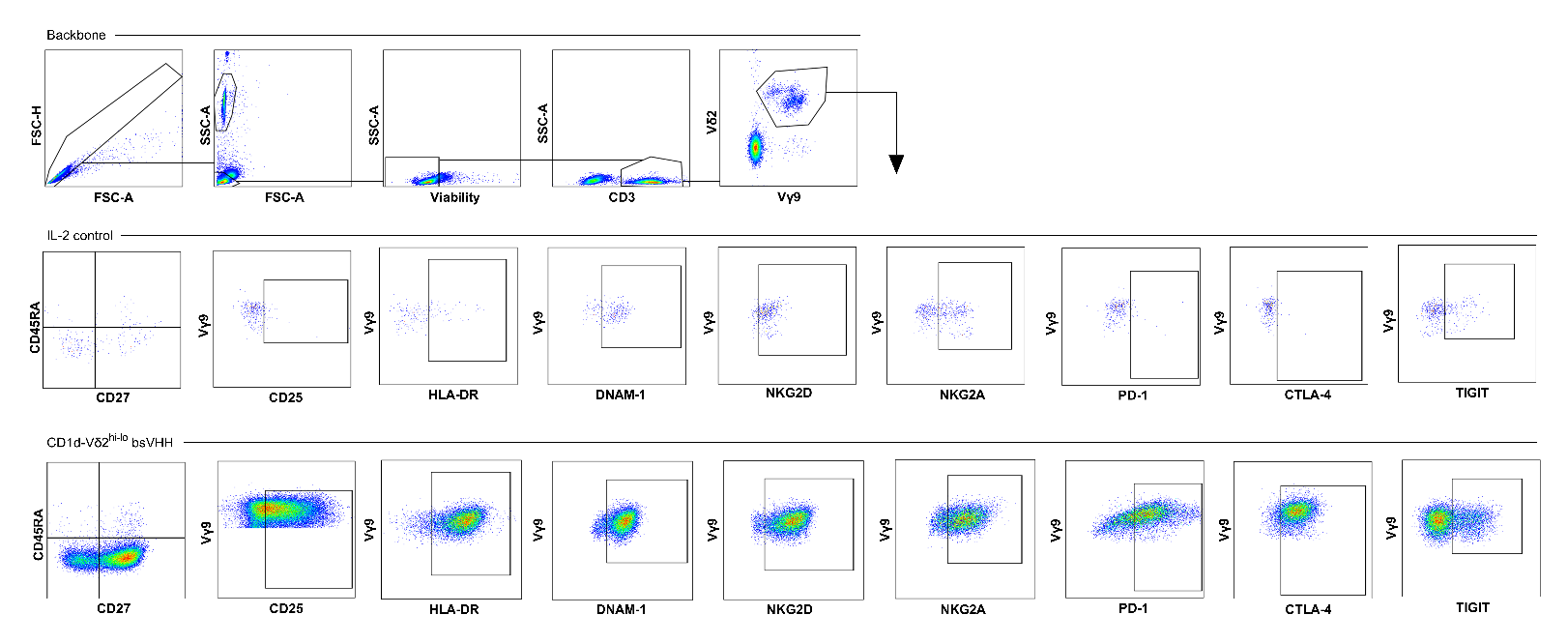
**

**Figure S6.**

Gating strategy used to analyze the phenotype of Vγ9Vδ2 T-cells in 8 day cultures of healthy donor- and cancer patient-derived PBMC supplemented with pamidronate, bivalent Vδ2^hi-lo^ VHH, TAA-Vδ2^hi-lo^ bsVHH and TAA-Vδ2^hi-lo^ bsVHH-Fc, or IL-2 control.

**
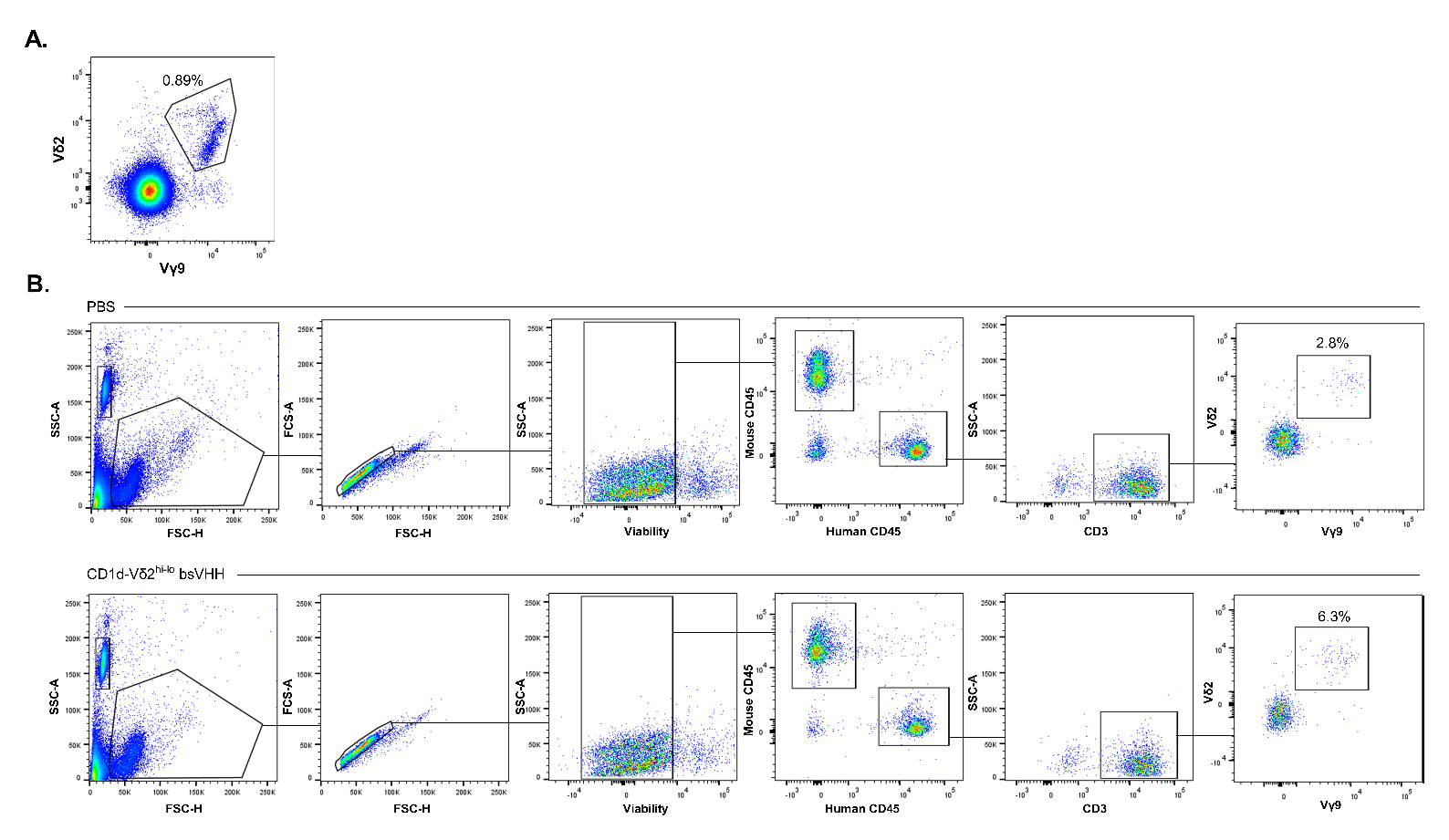
Figure S7.**

.(**A**) Vγ9Vδ2 T-cell frequency (of total CD3^+^ cells) of healthy human donor-derived PBMC used to inoculate NOG-hIL-15 mice. (**B**) Gating strategy used to analyze human PBMC-derived Vγ9Vδ2 T-cell frequencies and absolute counts 8 days after PBMC inoculation and two doses of i.p. injection with PBS or CD1d-Vδ2^hi-lo^ bsVHH in NOG-hIL-15 mice (shown are PBS and CD1d-Vδ2^hi-lo^ bsVHH in peripheral blood).
