# Supplementary material for: Vδ2 T-cell engagers bivalent for Vδ2-TCR binding provide anti-tumor immunity and support robust Vγ9Vδ2 T-cell expansion
Source: Front Oncol. 2024 Oct 18;14:1474007. doi: 10.3389/fonc.2024.1474007 (PMC11527600; doi:10.3389/fonc.2024.1474007)
Supplement: Supplementary file 2 [file Table1.docx]

| **Table S1. Details flow cytometry antibodies and viability dyes** | | | | | |
| --- | --- | --- | --- | --- | --- |
| **Marker** | **Fluorochrome** | **Clone** | **Catalogue number** | **Company** | **Host** |
| Anti-llama | FITC | Polyclonal | BET A160-100F | Bioke | Goat |
| CD3 | BV421 | SK7 | 563798 | BD horizon | Mouse |
| CD3 | PerCp-Cy5.5 | SK7 | 344808 | Biolegend | Mouse |
| CD25 | APC | 2A3 | 340907 | BD Biosciences | Mouse |
| CD25 | BV650 | M-A251 | 563718 | BD Biosciences | Mouse |
| CD27 | Pe-Cy7 | M-T271 | 560609 | BD Biosciences | Mouse |
| CD45 (human) | AF700 | HI30 | 304024 | Biolegend | Mouse |
| CD45 (mouse) | BV510 | 30-F11 | 563891 | BD Biosciences | Rat |
| CD45RA | APC-Cy7 | HI100 | 560674 | BD Pharmingen | Mouse |
| CD69 | PE | FN50 | 555531 | BD pharmingen | Mouse |
| CD107a | PE | eBioH4A3 | 12-1079-42 | eBioscience | Mouse |
| CTLA-4 | PE-CF594 | BNI3 | 562742 | BD Horizon | Mouse |
| DNAM-1 | FITC | 11A8 | 338304 | Biolegend | Mouse |
| HLA-DR | BV785 | L243 | 307642 | Biolegend | Mouse |
| NKG2A | BV421 | 131411 | 747924 | BD Biosciences | Mouse |
| NKG2D | APC | 1D11 | 558071 | BD Pharmingen | Mouse |
| PD-1 | BV786 | 563789 | EH12.1 | BD Horizon | Mouse |
| TIGIT | Pe-Cy7 | A15153G | 372713 | Biolegend | Mouse |
| Vδ2-TCR | FITC | IMMU389 | PN IM1464 | Beckman Coulter | Mouse |
| Vδ2-TCR | BV711 | B6 | 331412 | Biolegend | Mouse |
| Vγ9-TCR | APC | B3 | 331310 | Biolegend | Mouse |
| Vγ9-TCR | BV510 | B3 | 744035 | BD Biosciences | Mouse |
| Vγ9-TCR | PE | B3 | 331308 | Biolegend | Mouse |
| 7-AAD | PerCP-Cy5.5 | NA | A9400 | Sigma | N.A. |
| eFluor™ 506 | BV510 | NA | 65-0866 | eBioscience | N.A. |
| eFluor™ 780 | APC-CH7 | NA | 65-0865 | eBioscience | N.A. |

| **Table S2. Patient characteristics of donors used in correlation analyses** | | | | | | | |
| --- | --- | --- | --- | --- | --- | --- | --- |
| **Cancer type** | **Prostate**  **cancer** | **Esophageal cancer** | **Colorectal cancer** | **Melanoma** | **Gastric cancer** | **PDAC** | **Bladder cancer** |
| **n** | 40 | 26 | 8 | 7 | 5 | 4 | 1 |
| **Female,** n (%) | 0 (0%) | 5 (19%) | 3 (38%) | 2 (28%) | 3 (60%) | 2 (50%) | 0 |
| **Age, years** Mean (range) | 66 (39-77) | 68 (45-80) | 60 (42-77) | 73 (65-79) | 62 (48-78) | 64 (54-71) | 78 |
| **Stage,** n (%) I II  III  IV  Unknown | 25 (63%)  9 (23%)  4 (10%)  2 (5%) | 3 (12%)  6 (23%)  6 (23%)  1 (4%)  10 (38%) | 1 (13%)  2 (25%)  4 (50%)  1 (13%) | 5 (71%)  2 (29%) | 1 (20%)  1 (20%)  3 (60%) | 2 (50%)  2 (50%) | 1 |
| **Treatment,** n (%)  Chemotherapy  Radiotherapy Chemoradiotherapy  Checkpoint inhibition Naïve | 40 (100%) | 1 (4%)  25 (96%) | 5 (63%)  1 (13%)  2 (25%) | 4 (57%)  3 (43%) | 5 (100%) | 3 (75%)  1 (25%) | 1 |
| **Vγ9Vδ2 T-cells of CD3^+^ cells in peripheral blood at baseline, %** Mean (range) | 2.7%  (0.02-23.8) | 2.1%  (0.4-9.6) | 1.7% (0.1-7.8) | 1.8%  (0.2-5.1) | 2.3%  (0.3-5.7) | 1.7%  (0.2-4.5) | 0.5% |
| PDAC = Pancreatic ductal adenocarcinoma | | | | | | | |

| **Table S3. Patient characteristics of donors used in expansion assays** | | | | |
| --- | --- | --- | --- | --- |
| **Cancer type** | **Gastric cancer** | **Esophageal cancer** | **Melanoma** |  |
| **n** | 5 | 1 | 4 |  |
| **Female,** n (%) | 3 (60%) | 0 | 1 (25%) |  |
| **Age, years** Mean (range) | 62 (48-78) | 80 | 73 (67-77) |  |
| **Stage,** n (%) I II  III  IV | 1 (20%) 1 (20%) 3 (60%) | 1 | 3 (75%) 1 (25%) |  |
| **Treatment,** n (%)  Checkpoint inhibition Naïve | 5 (100%) | 1 | 2 (50%) 2 (50%) |  |
| **Vγ9Vδ2 T-cells of CD3^+^ cells in peripheral blood at baseline, %** Mean (range) | 2.31% (0.31-5.66) | 0.73% | 2.54% (1.03-4.47) |  |
